# *Helicobacter pylori* CagA promotes gastric cancer immune escape by upregulating SQLE

**DOI:** 10.1038/s41419-024-07318-w

**Published:** 2025-01-14

**Authors:** Sifan Liu, Nan Zhang, Xu Ji, Shuyue Yang, Zheng Zhao, Peng Li

**Affiliations:** Department of Gastroenterology, Beijing Friendship Hospital, Capital Medical University, State Key Laboratory for Digestive Health, National Clinical Research Center of Digestive Diseases, Beijing Digestive Disease Center, Beijing, 100050 China

**Keywords:** Gastric cancer, Oncogenes

## Abstract

*Helicobacter pylori* (*H. pylori*) infection is a well-established risk factor for gastric cancer, primarily due to its virulence factor, cytotoxin-associated gene A (CagA). Although PD-L1/PD-1-mediated immune evasion is critical in cancer development, the impact of CagA on PD-L1 regulation remains unclear. This study revealed that *H. pylori* CagA upregulated squalene epoxidase (SQLE) expression, a key enzyme in the cholesterol biosynthesis pathway. Elevated SQLE activity increased cellular palmitoyl-CoA levels, enhancing PD-L1 palmitoylation while decreasing its ubiquitination. This ultimately increases PD-L1 stability, suppressing T cell activity and facilitating immune evasion in gastric cancer. In summary, our findings highlight the crucial role of the CagA-SQLE-PD-L1 axis in gastric cancer progression, suggesting potential therapeutic strategies for targeting CagA-positive gastric cancer.

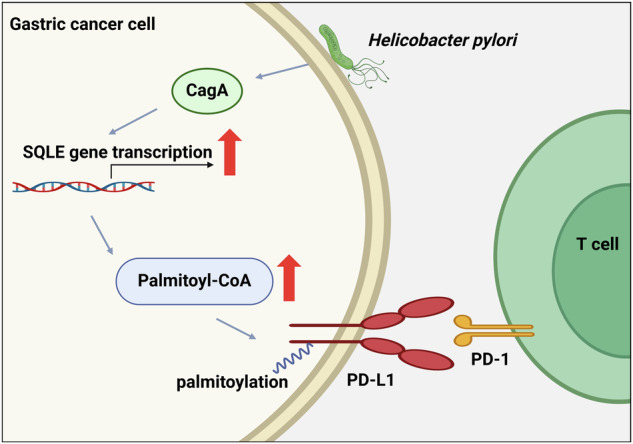

## Introduction

Gastric cancer is a major global health concern, ranking fifth in both incidence and mortality among cancers [1]. Gastric cancer is frequently diagnosed at advanced stages, resulting in poor prognosis. Despite chemotherapy’s status as a standard treatment, current agents yield a median survival time of only 8 months [2]. *H. pylori* infection is classified as a Group I carcinogen for gastric cancer by the World Health Organization [3]. Its role in the immune microenvironment is gaining attention, as *H. pylori* infection induces local inflammation and creates an immune-suppressive milieu [4]. Clinical retrospective studies have shown that *H. pylori* infection weakens the efficacy of immunotherapy in gastric cancer and non-small cell lung cancer [5–7]. Understanding the molecular mechanisms underlying *H. pylori*-induced immune tolerance is essential for optimizing gastric cancer treatment regimens.

Programmed death ligand 1 (PD-L1) serves as a crucial immune checkpoint by binding to programmed death 1 (PD-1) on T cells, resulting in decreased T cell activation [8]. CagA is a key virulence factor of *H. pylori* and contributes to the carcinogenic process by being injected into gastric epithelial cells via integrin receptors [9]. CagA upregulates PD-L1 transcription via the sonic hedgehog and p53-miR-34a pathways [10, 11]. In addition to transcriptional regulation, PD-L1 undergoes various post-translational modifications that affect its stability [12]. However, the influence of *H. pylori* CagA on PD-L1 stability remains unclear.

Transcriptome sequencing data revealed a substantial increase in squalene epoxidase (SQLE) expression in CagA-positive gastric cancer cells. SQLE catalyzes the conversion of squalene to (S)-2,3-oxidosqualene, a pivotal step in cholesterol biosynthesis [13]. It plays a significant role in various tumors, including colorectal cancer, pancreatic cancer, hepatocellular carcinoma, prostate cancer, and glioblastoma [14–18]. SQLE promotes hepatocellular carcinoma [19] and pancreatic cancer [14] through TGF-β/SMAD and Src/PI3K/Akt signaling pathways. It also represents a critical therapeutic target in advanced prostate cancer [20] and in colorectal cancer patients with p53 and c-MYC mutations [21]. However, the specific role of SQLE in gastric cancer remains to be elucidated. Studies have indicated that cholesterol can stabilize PD-L1 levels and prevent degradation by directly interacting with its transmembrane domain [22]. Given that SQLE plays a critical role in maintaining cholesterol levels, we hypothesized that SQLE may influence PD-L1 expression in gastric cancer.

The Hippo tumor suppressor pathway is crucial for maintaining tissue homeostasis and modulating immune responses [23]. YAP1 (Yes-Associated Protein 1) serves as a key downstream effector of this pathway [24]. Once phosphorylated, YAP1 binds to cytoskeletal proteins, resulting in its cytoplasmic sequestration [25, 26]. Conversely, unphosphorylated YAP1 translocated to the nucleus and promoted downstream oncogenes expression [27]. Previous study demonstrated that *H. pylori* CagA enhanced YAP1 protein expression and nuclear translocation, thereby promoting epithelial-mesenchymal transition and exerting oncogenic effects [26].

This study aims to elucidate the mechanisms through which *H. pylori* CagA modulates SQLE expression and assesses the contributions of CagA and SQLE to gastric cancer progression. Additionally, our findings indicate that CagA and SQLE enhance PD-L1 palmitoylation levels, leading to increased PD-L1 stability and suppression of T cell function, ultimately facilitating immune evasion in gastric cancer.

## Materials and methods

### Public transcriptome and RNA-sequencing analysis

Gastric cancer-related cohort data were obtained from the Gene Expression Omnibus (GEO) and The Cancer Genome Atlas (TCGA) databases. RNA-sequencing analysis was conducted on gastric cancer cells transfected with CagA at BGI (Shenzhen, China). GO pathway analysis was utilized to identify the most relevant pathways.

### Cell culture and transfection

Human and mouse gastric cancer cell lines, AGS (iCell-h016) and MFC (iCell-m035), were purchased from iCell Bioscience Inc. (Shanghai, China). Jurkat T cell lines were obtained from the laboratory stock. All cell lines have undergone STR identification within the last six months. AGS and MFC cell lines were cultivated in D/F-12 K medium and RPMI 1640 (Gibco, Waltham, MA, USA). These media formulations were augmented with 10% fetal bovine serum (FBS, Gibco), and the cell cultures were maintained in a 37 °C incubator under 5% CO_2_. Jurkat T cell lines were cultivated in RPMI 1640 (Gibco, Waltham, MA, USA) with 10% FBS, and maintained in a 37 °C incubator under 5% CO_2_. The universal mycoplasma detection kit (C0297S, Beyotime Biotechnology) was used to assess mycoplasma contamination at bi-monthly intervals.

The CagA-pcDNA 3.1 plasmid was purchased from Novobio Science (Shanghai, China), and the CagA sequence fragment was cloned into the p3 x Flag-CMV-10 vector. SQLE overexpression plasmids, HA-PD-L1 wild-type and the C272A mutant plasmids were procured from Youbio (Changsha, China). The HA-Ub plasmid was obtained from the laboratory stock. Short hairpin RNA (sh-RNA) targeting SQLE, and small interfering RNA (si-RNA) targeting YAP1 were purchased from GenePharma (Shanghai, China). The sequences of sh-SQLE and si-YAP1 used for transfection are shown in Table S1. Transfection was carried out with Lipofectamine 2000 (11668019, Thermo Fisher Scientific) or Megatran 2.0 (TT210003, Origene).

### *H. pylori* culture and infection

*H. pylori* strain 26695 was sourced from laboratory stock. The *H. pylori* strains were cultured at 37 °C in a microaerophilic atmosphere on a Columbia agar plate (Oxoid Ltd, Basingstoke, UK) which contained 10% sheep’s blood.

In *H. pylori* infection assays, AGS and MFC cells were cultured in six-well plates containing antibiotic-free medium for 24 h and subsequently infected with *H. pylori* at a multiplicity of infection (MOI) of 15. 24 h later, cells were collected for subsequent experiments.

### Nucleic acid gel electrophoresis

PCR assay and gel electrophoresis were performed to identify *H. pylori* strains. The primer sequences used were shown in Table S1. The PCR reaction system (20 μL) included 2 × PCR Master Mix 10 μL, 0.75 μL upstream primer, 0.75 μL downstream primer, 2 μL bacteria solution, and 6.5 μL with ddH_2_O. The reaction condition was as follows. 23 s rRNA: 95 °C 2 min; 4 °C 30 s, 60 °C 30 s, 72 °C 30 s, 40 cycles; 72 °C 5 min; 4 °C maintained. CagA: 95 °C 2 min; 94 °C 30 s, 54 °C 30 s, 72 °C 30 s, 40 cycles; 72 °C 5 min; 4 °C maintained. Vacuolating cytotoxin A (VacA): 95 °C 2 min; 94 °C 30 s, 55 °C 30 s, 72 °C 30 s, 40 cycles; 72 °C 5 min; 4 °C maintained. At the end of the reaction, 1% gel electrophoresis was performed. The size of the amplified target fragment was 490 bp (23s rRNA), 350 bp (CagA), and 250 bp (VacA). The experiment was repeated 3 times at least.

### Reagents

Verteporfin (HY-B0146), cycloheximide (CHX, HY-12320), MG132 (HY-13259), and methyl-β-cyclodextrin (MCD, HY-101461) were obtained from MedChemExpress. Palmitoyl coenzyme A (Pal-CoA, P9716) and cholesterol (C8667) were purchased from Merck Sigma. 2-bromopalmitate (2-BP, E0120) was obtained from Selleck.

### Cholesterol concentration determination

The total cholesterol assay kit (RL7528-100T) based on the COD-PAP single reagent colorimetric method was procured from BioRoYee (Beijing, China) and utilized to detect total cholesterol in transfected gastric cancer cells.

### RNA extraction and reverse transcription-quantitative polymerase chain reaction (qPCR)

Total RNA was extracted from cells with the TRIzol reagent (15596018, Thermo Fisher Scientific), and reverse transcription reactions were performed with the PrimeScript™ RT Master Mix (RR036A, TaKaRa). qPCR was performed using Fast SYBR Green Master Mix (A25742, Thermo Fisher Scientific). ACTB and GAPDH served as internal controls. Quantification was calculated using the 2^−∆∆Ct^ method. The synthesis of primers was entrusted to Sangon Biotech (Shanghai, China), and the corresponding primer sequences can be found in Table S1.

### Protein extraction and western blotting (WB)

Cells were lysed in RIPA Lysis Buffer (P0013C, Beyotime Biotechnology) supplemented with protease and phosphatase inhibitors. Protein concentrations were measured with the BCA reagent (BL521A, Biosharp). The protein lysate was separated by sodium dodecyl sulfate (SDS)-PAGE gel and then transferred to a polyvinylidene difluoride membrane. After blocking with non-fat milk for 2 h at room temperature, the membranes were incubated overnight at 4 °C with the following primary antibodies: anti-SQLE (1:1000, 12544-1-AP, Proteintech), anti-PD-L1 (1:4000, 66248-1-Ig, Proteintech), anti-YAP1 (1:1000, A26076, ABclonal), anti-Vinculin (1:20000, 26520-1-AP, Proteintech), anti-Flag (1:1000, F1804, Merck Sigma), anti-phosphor-YAP1-S127 (1:200, AP0489, ABclonal), anti-phosphor-YAP1-S128 (1:3000, AP1187, ABclonal), anti-β-Tubulin (1:2000, AF1216, Beyotime), anti-HA (1:2000, TA180218, Origene), anti-CagA (1:200, sc-28368, Santa Cruz Biotechnolog), anti-LAG3 (1:1000, 16616-1-AP, Proteintech), anti-TIM3 (1:1000, 60355-1-Ig, Proteintech), and anti-CTLA-4 (1:5000, A22865, ABclonal). After washing, the membranes were stained with secondary antibodies for 1.5 h at room temperature. Protein bands were detected using the ChemiDoc XRS+ system (Bio-rad, USA).

### Cell counting kit-8 (CCK-8) assay

Transfected gastric cancer cells were planted into 96-well plates at a density of 1 × 10^3^ cells per well, with 4-6 replicate wells per group. At specified time points, the medium was replaced with 100 μl fresh culture medium containing 10 μl CCK-8 solution (C0039, Beyotime Biotechnology), incubated at 37 °C for 2 h. Then the absorbance at 450 nm was detected.

### Colony formation assay

Transfected cells were seeded into 3.5 cm dishes at a density of 500 cells per dish. The experiment was terminated upon visible clone formation. Cells were fixed with methanol and stained with 0.5% crystal violet solution for 15 min. Colonies were counted and images were acquired. Colonies with a cell count exceeding 50 were considered significant.

### Wound healing assay

Transfected cells were seeded in six-well plates and allowed to reach confluence. Subsequently, wounds were created in the monolayers using sterile plastic implements. After phosphate buffer saline (PBS) wash, the cells were cultured in medium containing 0.5% FBS. Cellular migration was evaluated by capturing images at 0, 12, 24, 36, and 48 h. The wound area was analyzed using ImageJ software.

### Transwell assay

Transwell assay was performed in a 24-well Transwell insert with an 8 μm pore size (353097, Falcon Corning). Transfected gastric cancer cells were seeded into the upper chambers without FBS at a density of 2.5 × 10^4^, while the lower chambers were filled with 750 µL of cell culture medium supplemented with 10% FBS. After incubation at 37 °C for 36 h, the non-migratory gastric cancer cells remaining inside the upper chambers were removed with cotton swabs, while the cells on the lower surface of the membrane were fixed, stained, and then counted under a light microscope. Cell counting was performed using ImageJ software.

### T cell-mediated tumor cell killing assay

Transfected AGS cells adhered to plates overnight before co-incubating with Jurkat T cells for 48 h at a ratio of 1:10 (AGS cells: T cells). Following PBS washes to remove Jurkat T cells and debris, live gastric cancer cells were stained with 0.5% crystal violet and quantified using a spectrophotometer at OD 570 nm.

### T cell activity assays

Transfected AGS cells were seeded on the glass bottom, light-resistant 3.5 cm dishes for 48 h, subjected to staining with the live-cell fluorescent dye DiD perchlorate (HY-D1028, MedChemExpress), and co-cultured with Jurkat T cells labeled with the Green CMFDA (HY-126561, MedChemExpress) at a ratio of 1:10. Continuous imaging was performed using a high-resolution imaging system (DeltaVision Ultra) to track the activity status of living cells.

### Immunohistochemistry (IHC) staining

IHC was performed following the standard protocol: after xylene and graded ethanol treatment, the sections were subjected to antigen retrieval by boiling in either an EDTA solution (pH = 8.0) or a citrate solution. Then the sections were blocked and incubated with the following antibodies: anti-*H. pylori* (ZA-0127, Origene), anti-CagA (1:100, sc-28368, Santa Cruz), anti-SQLE (1:50, 12544-1-AP, Proteintech), and anti-PD-L1 (1:5000, 66248-1-Ig, Proteintech) respectively, overnight at 4 °C in a humidified chamber, subsequently incubated with HRP-conjugated secondary antibodies for 2 h. Immunodetection was visualized with diaminobenzidine (DAB) for 3-5 min, then the sections were counterstained with hematoxylin. The use of patient pathological tissue sections was approved by the Medical Ethical Committee of the Beijing Friendship Hospital Affiliated to the Capital Medical University (2018-P2-058).

### Co-immunoprecipitation (Co-IP) assay

Cells were lysed in IP buffer (P0013, Beyotime Biotechnology) containing protease and phosphatase inhibitors on ice for 25 min, followed by centrifugation at 12,000 rpm for 15 min at 4 °C to remove insoluble materials. 3 μg of the indicated antibody was added into the supernatant and incubated at 4 °C overnight. Then, Protein A/G beads (P2108, Beyotime Biotechnology) were added and incubated for 2 h at room temperature. The beads were washed with IP buffer three times and eluted by boiling with SDS buffer. The precipitated components were detected by WB.

### Click-iT assay

Transfected cells were incubated with 100 μM of Click-iT palmitic acid-azide (HY-151656, MedChemExpress) for 6 h. After incubation, cells were lysed with the lysis buffer containing 1% SDS in 50 mM Tris-HCl, pH 8.0, with protease and phosphatase inhibitors, then left on ice for 15 min. Click-iT Protein Reaction Buffer Kit (C10276, Thermo Fisher Scientific) was used to catalyze the reaction of protein samples with biotin-alkyne. Subsequently, the biotin alkyne-azide-palmitic protein complex was pulled down using washed streptavidin (3419, Cell Signaling Technology). The resulting pellets were then analyzed by immunoblotting to detect PD-L1 expressions.

### Elisa assay

The human palmitoyl Coenzyme A ELISA kit (JLCA7899) was procured from Jingkang (Shanghai, China) and utilized to detect palmitoyl-CoA.

### Animal studies

All mice used in this study were supplied by and maintained under specific pathogen-free conditions at Capital Medical University (Beijing, China). Six-week-old male BALB/c nude mice and C57BL/6 mice were purchased from Beijing Vital River Laboratory Animal Technology Co., Ltd and used for tumorigenesis. The mice were randomly divided into 3 groups (*n* = 5) respectively. BALB/c nude mice and C57BL/6 mice were subcutaneously injected with equal amounts of stably transfected MFC cells (3.5 × 10^6^) to initiate tumorigenesis. Tumor volume was measured using the formula: length × width^2^/2. The mice were sacrificed 12 days after inoculation. The tumor volume and weight were measured and analyzed. The animal experiments conducted in this project were approved and reviewed by the Animal Experiments and Experimental Animal Welfare Committee of Capital Medical University (AEEI-2023-098).

### Statistical analysis

Results are expressed as mean ± SD based on a minimum of three independent experiments. The sample size was determined based on preliminary data showing the variance within and between groups. Statistical analyses were conducted using GraphPad Prism version 8 and ImageJ version 1.37c. For multi-group comparisons, analysis of variance (ANOVA) was employed, while the two-sided student *t*-tests were utilized for pairwise comparisons. Statistical significance was established at a *P*-value less than 0.05.

## Results

### *H. pylori* CagA overexpression correlates with elevated SQLE expression

**Fig. 1:**
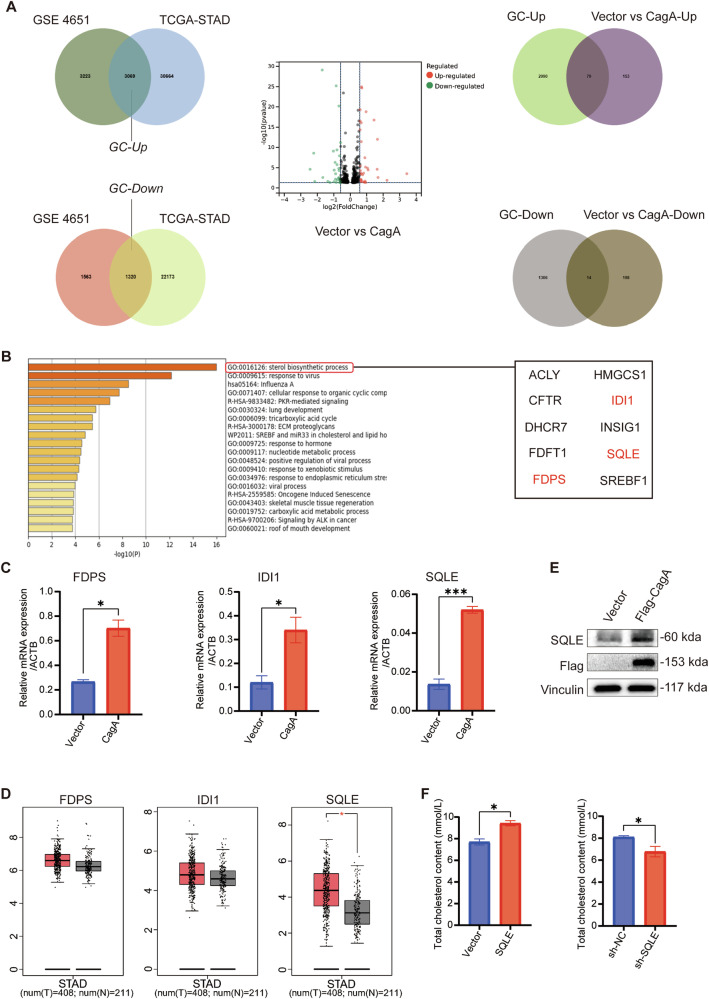
SQLE was highly expressed in gastric cancer cells overexpressing CagA. **A** Key gene screening: gastric cancer-related differential genes were obtained from GEO and TCGA public databases and intersected with vector vs CagA. **B** GO analysis of 93 differentially expressed genes. **C** mRNA levels of FDPS, IDI1, and SQLE were examined after CagA overexpression in AGS. **D** mRNA levels of FDPS, IDI1, and SQLE in tumor and normal tissues were analyzed using TCGA database. **E** WB was performed to quantify SQLE protein level after Flag-CagA overexpression in AGS. Vinculin was used as an internal control. **F** COD-PAP single reagent colorimetric was used to detect the content of intracellular total cholesterol after SQLE overexpression or knockdown in AGS. Data are presented as mean ± SD. **** *P* < 0.0001; *** *P* < 0.001; ** *P* < 0.01; * *P* < 0.05; ns *P* > 0.05.

CagA, a significant virulence factor of *H. pylori*, plays a crucial role in the initiation and progression of gastric cancer [28]. To further investigate its contribution, we identified differentially expressed genes in gastric cancer compared to normal tissues utilizing publicly available databases. Cross-referencing these genes with those regulated by CagA in gastric cancer cell lines (Table S2) revealed 79 up-regulated genes and 14 down-regulated genes (Fig. 1A). Subsequent enrichment analysis indicates that the sterol biosynthetic process pathway, as depicted in Fig. 1B, displays the most pronounced variation. Within this pathway, the roles of farnesyl diphosphate synthase (FDPS), isopentenyl-diphosphate delta isomerase 1 (IDI1), and SQLE in gastric cancer remain largely unexplored. To gain deeper insights, we conducted qPCR experiments to measure their expression levels in CagA-overexpressed gastric cancer cells (Fig. 1C) and analyzed their expression based on TCGA public database (Fig. 1D). Notably, SQLE exhibited the most significant difference, suggesting its potential role as a downstream gene of CagA and its pivotal role in gastric cancer progression.

To further confirm the influence of CagA on SQLE expression, we overexpressed Flag-CagA in the gastric cancer cell line AGS and observed a significant increase in both SQLE protein and mRNA levels compared to control cells (Figs. 1E and S1A). Similar results were observed in MFC cells (Fig. S1B, C). Moreover, we conducted co-culture experiments using *H. pylori* 26695 strains (CagA-positive or CagA-negative) with gastric cancer cells (Fig. S1D, E). qPCR and WB assays demonstrated that gastric cancer cells co-cultured with CagA-positive *H. pylori* strains exhibited elevated expression of SQLE (Fig. S1F–J). Conversely, no changes were observed in gastric cancer cells co-cultured with CagA-negative *H. pylori* (Fig. S1K–N). Thus, we conclude that CagA can indeed promote SQLE expression in gastric cancer.

Furthermore, to substantiate the involvement of SQLE in regulating cholesterol metabolism, we established an SQLE-knockdown AGS cell line (Fig. S1O–Q) and evaluated intracellular total cholesterol levels after SQLE overexpression or knockdown. Our results demonstrated a positive correlation between SQLE expression levels and intracellular total cholesterol (Fig. 1F), indicating the participation of SQLE in cholesterol metabolism.

### CagA induces SQLE expression via a YAP1-mediated mechanism

**Fig. 2:**
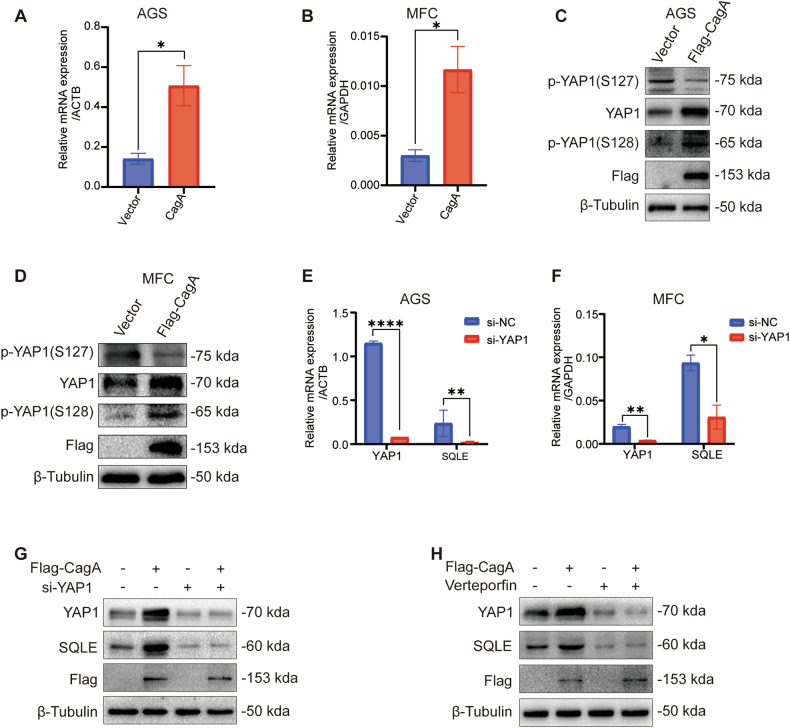
*H. pylori* CagA regulates SQLE expression through YAP1. mRNA levels of YAP1 were examined after CagA overexpression in AGS (**A**) and MFC (**B**). WB was performed to quantify the protein levels of YAP1 and p-YAP1 after Flag-CagA overexpression in AGS (**C**) and MFC (**D**). β-Tubulin was used as an internal control. mRNA levels of YAP1 and SQLE were evaluated after YAP1 knockdown in AGS (**E**) and MFC (**F**). **G** WB was employed to detect SQLE protein levels in AGS cells after transfection with si-YAP1 for 24 h and overexpression of Flag-CagA for 48 h. β-Tubulin was used as an internal control. **H** WB was employed to assess SQLE protein level following treatment with the YAP1 inhibitor verteporfin (10 mM, 24 h) and Flag-CagA overexpression for 48 h in AGS cells. β-Tubulin was used as an internal control. Data are presented as mean ± SD. **** *P* < 0.0001; *** *P* < 0.001; ** *P* < 0.01; * *P* < 0.05; ns *P* > 0.05.

Research indicates a strong association between *H. pylori* infection and the Hippo-YAP1 pathway in gastric cancer [26]. Thus, we investigated the effect of CagA on YAP1 expression. We observed that CagA overexpression in AGS and MFC cell lines led to variable increases in both mRNA (Fig. 2A, B) and protein (Figs. 2C, D and S2A) levels of YAP1. Furthermore, we noted that CagA overexpression resulted in elevated expression of cancer-promoting phosphorylated-YAP1 (S128) and decreased expression of cancer-inhibiting phosphorylated-YAP1 (S127) (Figs. 2C, D and S2A). These findings suggest that CagA overexpression in gastric cancer influences YAP1-mediated signaling axis.

To investigate whether CagA regulates SQLE expression via YAP1, we conducted YAP1 knockdown experiments, observing a simultaneous decrease in SQLE expression at both mRNA (Fig. 2E, F) and protein levels (Figs. 2G and S2B). Treatment with si-YAP1 (Figs. 2G and S2B) and the YAP1 inhibitor verteporfin (Figs. 2H and S2C) effectively suppressed SQLE expression. However, subsequent overexpression of CagA did not further enhance the elevation of SQLE. These findings suggest that CagA modulates SQLE expression through YAP1.

### CagA and SQLE promote the proliferation and migration of gastric cancer

**Fig. 3:**
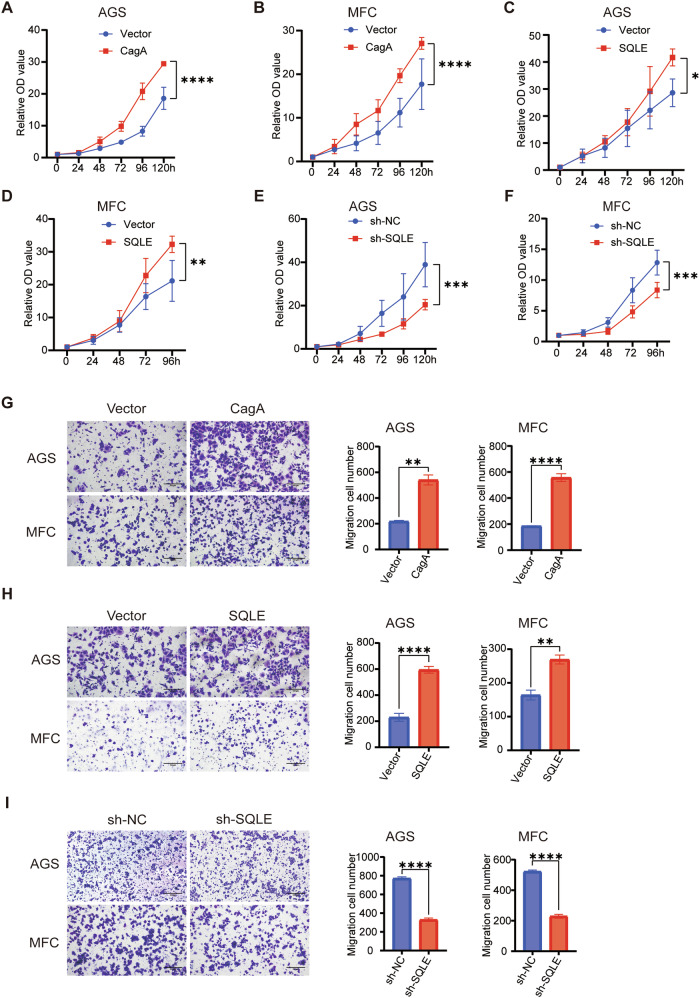
CagA and SQLE promote gastric cancer progression. The proliferation of AGS (**A**) and MFC (**B**) cells following CagA overexpression was assessed using the CCK-8 assays. CCK-8 assays were used to examine the proliferation of AGS (**C**) and MFC (**D**) cells after SQLE overexpression. CCK-8 assays were used to evaluate the proliferation of AGS (**E**) and MFC (**F**) cells after SQLE knockdown. **G** Left, transwell assays were used to determine the migration of AGS and MFC after CagA overexpression. Right, quantitative analysis results of it (*n* = 3). Scale bar = 100 μm. **H** Left, transwell assays were used to detect the migration of AGS and MFC after SQLE overexpression. Right, quantitative analysis results of it (*n* = 3). Scale bar = 100 μm. **I** Left, transwell assays were used to identify the migration of AGS and MFC after SQLE knockdown. Right, quantitative analysis results of it (*n* = 3). Scale bar = 100 μm. Data are presented as mean ± SD. **** *P* < 0.0001; *** *P* < 0.001; ** *P* < 0.01; * *P* < 0.05; ns *P* > 0.05.

Next, we investigated the potential impact of CagA and SQLE on the malignant characteristics of gastric cancer. CCK-8 assays demonstrated that the overexpression of CagA (Fig. 3A, B) and SQLE (Fig. 3C, D) could enhance gastric cancer cell proliferation. Furthermore, we established an SQLE-knockdown MFC cell line (Fig. S3A–C) and observed that inhibiting SQLE reduced cell proliferation (Fig. 3E, F). Consistent results were obtained in colony formation assays (Fig. S3D–F). These findings underscore the role of CagA and SQLE in promoting gastric cancer cell proliferation.

Furthermore, transwell and wound healing assays were conducted to assess the migration ability of gastric cancer cells. The results indicated that the migration ability of AGS and MFC cells was enhanced in the CagA (Figs. 3G and S3G-H) and SQLE (Figs. 3H and S3I-J) overexpression groups compared to the vector group. Conversely, migration ability decreased following SQLE knockdown (Figs. 3I and S3K). These findings collectively suggest that *H. pylori* CagA and SQLE play a role in mediating gastric cancer proliferation and migration.

### CagA enhances PD-L1 expression via SQLE

**Fig. 4:**
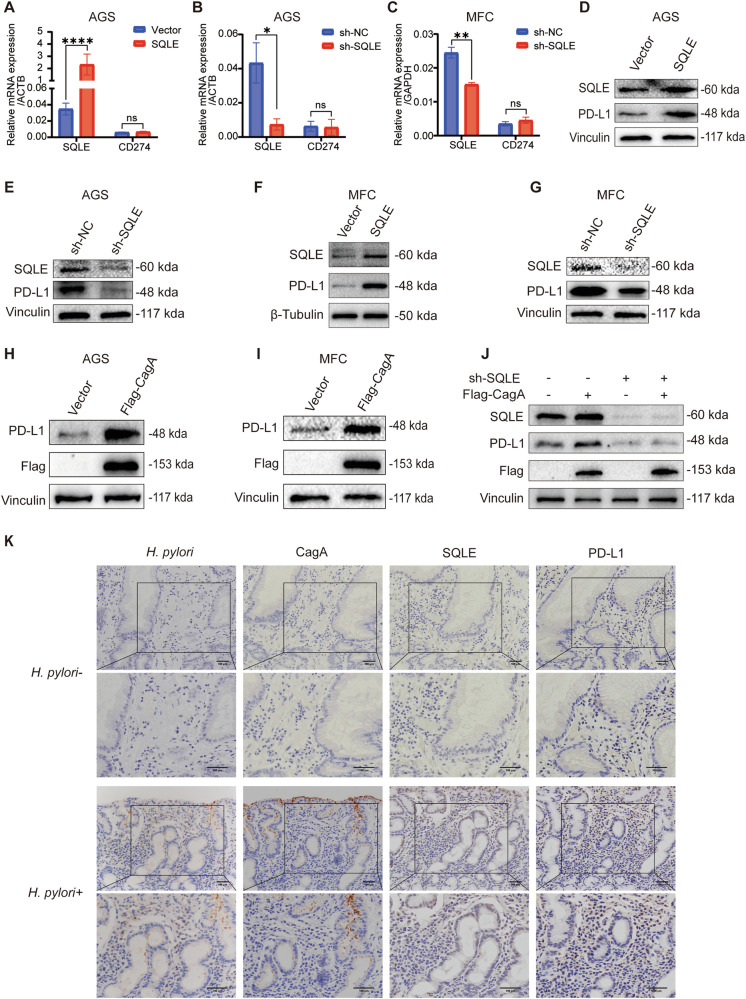
*H. pylori* CagA promotes PD-L1 expression through SQLE. **A** qPCR was used to detect the mRNA levels of SQLE and CD274 in AGS after SQLE overexpression. qPCR was performed to examine the mRNA levels of SQLE and CD274 in AGS (**B**) and MFC (**C**) after SQLE knockdown. **D** WB was used to identify PD-L1 protein levels after SQLE overexpression in AGS. Vinculin was used as an internal control. **E** WB was used to identify PD-L1 protein levels after SQLE knockdown in AGS, using vinculin as an internal control. **F** WB was used to identify PD-L1 protein levels after SQLE overexpression in MFC, using β-Tubulin as an internal control. **G** WB was used to identify PD-L1 protein levels after SQLE knockdown in MFC. Vinculin was used as an internal control. WB was used to detect PD-L1 protein levels after Flag-CagA overexpression in AGS (**H**) and MFC (**I**) cells. Vinculin was used as an internal control. **J** WB was used to detect PD-L1 protein levels after Flag-CagA overexpression in SQLE knockdown AGS cells. Vinculin was used as an internal control. **K** IHC staining images for *H. pylori*, CagA, SQLE, and PD-L1 in gastric cancer patients. Scale bar = 100 μm. Data are presented as mean ± SD. **** *P* < 0.0001; *** *P* < 0.001; ** *P* < 0.01; * *P* < 0.05; ns *P* > 0.05.

Recent research has demonstrated that cholesterol binds to the PD-L1 molecule CRAC motif, enhancing PD-L1 stability by inhibiting ubiquitination [22]. Thus, our study aims to explore the regulatory role of SQLE in PD-L1 levels. qPCR results revealed that altering SQLE expression did not significantly impact PD-L1 mRNA levels (Fig. 4A–C). However, a positive correlation was observed between PD-L1 protein levels and SQLE (Figs. 4D–G and S4A). CagA overexpression similarly facilitated PD-L1 protein expression (Fig. 4H–I). Furthermore, the CagA-positive *H. pylori* strain upregulated PD-L1 protein levels (Fig. S4B), while the CagA-negative strain had no effect on PD-L1 expression levels (Fig. S4C). Notably, downregulating SQLE hindered CagA-induced PD-L1 expression (Fig. 4J), suggesting a potential regulatory pathway of CagA on PD-L1 via SQLE.

The relationship among *H. pylori*, CagA, SQLE, and PD-L1 expression was further confirmed in clinical pathological specimens through IHC analysis, demonstrating a positive correlation (Fig. 4K). In conclusion, our findings suggest that *H. pylori* CagA promotes PD-L1 expression through SQLE in gastric cancer.

### SQLE promotes PD-L1 palmitoylation and inhibits its ubiquitination

**Fig. 5:**
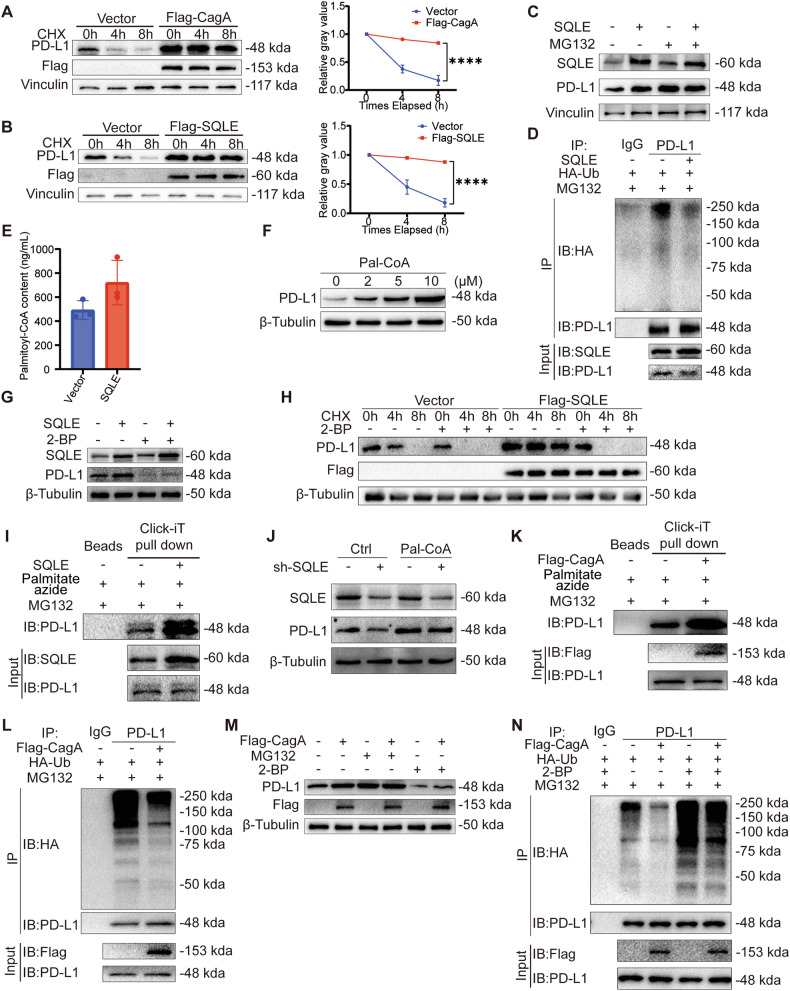
SQLE promotes PD-L1 palmitoylation and inhibits its ubiquitination. Left, CHX-chase assays were used to evaluate PD-L1 degradation in AGS cells overexpressing Flag-CagA (**A**) and Flag-SQLE (**B**), using vinculin as a control. Right, quantitative analysis results of it (*n* = 3). **C** WB was performed to detect PD-L1 protein levels in MFC cells overexpressing SQLE after MG132 (10 μM for 4 h) incubation, using vinculin as a control. **D** Co-IP was used to detect PD-L1 ubiquitination after MG132 (10 μM for 6 h) incubation in MFC cells overexpressing SQLE. **E** LC-MS/MS was used to detect palmitoyl-CoA content in MFC cells overexpressing SQLE. **F** WB was used to examine the protein level of PD-L1 in AGS after the use of Pal-CoA (0 to 10 μM for 48 h), using β-Tubulin as an internal control. **G** WB was used to identify PD-L1 protein levels in MFC cells overexpressing SQLE after 2-BP (100 μM for 24 h) incubation, using β-Tubulin as an internal control. **H** CHX-chase assay was used to demonstrate the 2-BP (100 μM for 24 h) effect on PD-L1 degradation in MFC cells overexpressing Flag-SQLE, using β-Tubulin as an internal control. **I** Click-iT reaction was used to detect PD-L1 palmitoylation in MFC cells overexpressing SQLE. Click-iT palmitate azide (100 μM for 6 h) and MG132 (10 μM for 6 h) were added before the samples were collected. **J** WB was used to detect PD-L1 protein levels after Pal-CoA (10 μM for 48 h) incubation in SQLE knockdown AGS cells, using β-Tubulin as a control. **K** Click-iT reaction was used to detect PD-L1 palmitoylation in MFC cells overexpressing Flag-CagA. Click-iT palmitate azide (100 μM for 6 h) and MG132 (10 μM for 6 h) were added before the samples were collected. **L** Co-IP was used to detect PD-L1 ubiquitination after MG132 (10 μM for 6 h) incubation in MFC cells overexpressing Flag-CagA. **M** WB was used to identify PD-L1 protein levels in MFC cells overexpressing Flag-CagA after the use of MG132 (10 μM for 6 h) or 2-BP (100 μM for 24 h), using β-Tubulin as a control. **N** Co-IP was used to identify PD-L1 ubiquitination after 2-BP (100 μM for 24 h) and MG132 (10 μM for 6 h) incubation in MFC cells overexpressing Flag-CagA. Data are presented as mean ± SD. **** *P* < 0.0001; *** *P* < 0.001; ** *P* < 0.01; * *P* < 0.05; ns *P* > 0.05.

Our findings indicated that CagA regulated PD-L1 expression at the protein level, while mRNA levels remained unaffected. To elucidate the mechanism of CagA-mediated PD-L1 regulation, we employed the protein synthesis inhibitor CHX. Our results showed that PD-L1 expression gradually declined upon CHX treatment in the vector group yet remained stable in the CagA overexpression group (Fig. 5A). Similar trends were observed with the SQLE overexpression (Fig. 5B), indicating a potential role for CagA in stabilizing PD-L1 structure.

Moreover, we investigated the impact of SQLE on PD-L1 ubiquitination. Treatment with the proteasome inhibitor MG132 yielded comparable PD-L1 expression levels between control and SQLE overexpression groups (Fig. 5C). Further analysis revealed a significant decrease in PD-L1 ubiquitination levels upon SQLE overexpression (Fig. 5D), suggesting SQLE stabilizes PD-L1 by inhibiting ubiquitination. To explore the mechanisms by which SQLE affects PD-L1 ubiquitination levels, we initially assessed the potential interaction between SQLE and PD-L1. Co-IP results indicated that SQLE does not interact with PD-L1 (Fig. S5A, B).

During cholesterol synthesis, various coenzyme A derivatives are generated. Hence, we performed a metabolomic analysis on gastric cancer cells overexpressing SQLE. The LC-MS/MS analysis revealed an elevated concentration of Pal-CoA, the substrate for palmitoylation, in cells overexpressing SQLE (Fig. 5E). We next investigated the effect of Pal-CoA on PD-L1 palmitoylation. Increasing Pal-CoA concentration by adding Pal-CoA-lithium (Fig. S5C) led to a concentration-dependent increase in PD-L1 expression (Fig. 5F). Additionally, following Pal-CoA addition, Click-iT assay showed that PD-L1 palmitoylation markedly increased (Fig. S5D, E) and its ubiquitination significantly decreased (Fig. S5F). After inhibiting palmitoylation with the palmitoylation inhibitor 2-BP, PD-L1 ubiquitination levels increased in both the control group and Pal-CoA group. Furthermore, the inhibitory effect of Pal-CoA on PD-L1 ubiquitination was diminished (Fig. S5G), indicating that Pal-CoA enhances PD-L1 palmitoylation and suppresses its ubiquitination. These results suggest that Pal-CoA plays an important role in PD-L1 post-translational modifications.

Subsequently, we investigated the effect of SQLE on PD-L1 palmitoylation. Treating cells overexpressing SQLE with 2-BP resulted in decreased PD-L1 expression levels (Fig. 5G). Co-treatment with 2-BP and CHX in cells overexpressing SQLE showed reduced protein stability of PD-L1 following palmitoylation inhibition (Fig. 5H). Click-iT assay revealed higher palmitoylation upon SQLE overexpression compared to the vector group (Fig. 5I). Additionally, SQLE reduced PD-L1 ubiquitination levels. However, when PD-L1 palmitoylation was inhibited with 2-BP, the inhibitory effect of SQLE on PD-L1 ubiquitination was significantly diminished (Fig. S5H), indicating that SQLE promotes PD-L1 palmitoylation to inhibit its ubiquitination. SQLE inhibition led to decreased PD-L1 expression levels. However, when additional Pal-CoA was supplied, the effect of SQLE on PD-L1 expression was significantly reduced (Figs. 5J and S5I), suggesting that SQLE promotes PD-L1 expression through Pal-CoA. Furthermore, previous studies have identified Cys272 as the palmitoylation site of PD-L1 [29]. We also utilized a non-palmitoylatable mutant, PD-L1-C272A, and found that when Cys272 was mutated to alanine, PD-L1 palmitoylation was undetectable (Fig. S5J). This suggests that Cys272 is likely the only site of palmitoylation on PD-L1, with SQLE promoting PD-L1 palmitoylation at this residue.

Similarly, CagA overexpression increased PD-L1 palmitoylation level (Fig. 5K) and decreased its ubiquitination level (Fig. 5L). Furthermore, upon separately inhibiting ubiquitination and palmitoylation, CagA failed to promote PD-L1 expression (Fig. 5M). CagA overexpression reduced PD-L1 ubiquitination levels. However, when inhibiting palmitoylation with 2-BP, the effect of CagA on PD-L1 ubiquitination was significantly diminished (Fig. 5N). These findings collectively suggest that CagA and SQLE enhance PD-L1 stability by promoting its palmitoylation.

Cholesterol interacts directly with the transmembrane domain of PD-L1, preventing its degradation and stabilizing its levels [22]. In gastric cells, cholesterol supplementation increased PD-L1 levels, while treatment with the cholesterol-depleting agent MCD significantly reduced them (Fig. S5K, L). Cholesterol addition led to a marked decrease in PD-L1 ubiquitination, whereas MCD significantly increased it (Fig. S5M, N). However, Click-iT assays showed that cholesterol has a minimal effect on PD-L1 palmitoylation (Fig. S5O-P). Furthermore, SQLE promoted PD-L1 expression even when treated with MCD (Fig. S5Q). Therefore, we propose that SQLE influences PD-L1 stability by promoting cholesterol synthesis and increasing intracellular Pal-CoA, thereby enhancing PD-L1 palmitoylation and inhibiting its ubiquitination.

### CagA and SQLE diminished the tumor-killing capacity and activity of T cells

**Fig. 6:**
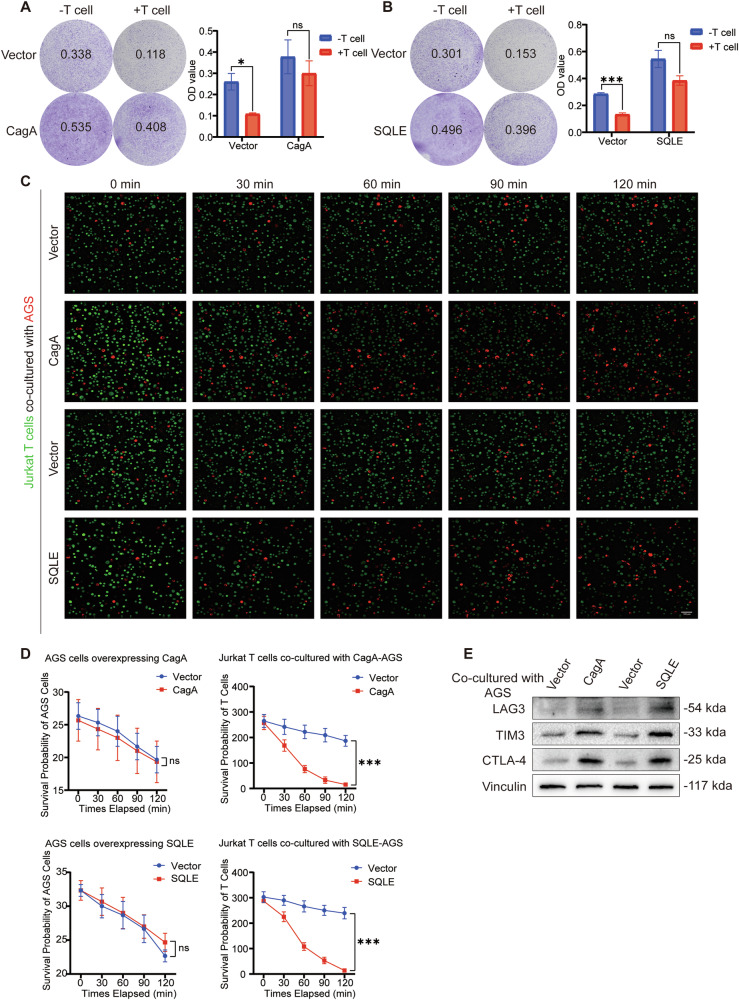
CagA and SQLE overexpression led to decreased T cell activity. Left, T cell-mediated cancer cell-killing assay results. AGS cells overexpressing CagA (**A**) and SQLE (**B**) co-cultured with Jurkat T cells for 48 h were subjected to crystal violet staining. The ratio of Jurkat T cell to AGS cell is 10:1. Right, the OD value of live cells in each well was determined at a wavelength of 570 nm. **C** A high-resolution live-cell imaging was performed to follow up the activity of Jurkat T cells co-cultured with AGS cells overexpressing CagA and SQLE; living tumor cells: red (DiD perchlorate); living Jurkat T cells: green (Green CMFDA). Scale bar = 100 μm. **D** The statistical analysis plot according to the AGS cell and Jurkat T cell signaling intensity was shown. Scale bar = 100 μm. **E** WB was used to detect the protein level of Jurkat T cell exhaustion markers (LAG3, TIM3, and CTLA-4) after co-culturing with AGS cells overexpressing CagA and SQLE. Vinculin was used as an internal control. Data are presented as mean ± SD. **** *P* < 0.0001; *** *P* < 0.001; ** *P* < 0.01; * *P* < 0.05; ns *P* > 0.05.

Previous findings have established that CagA enhances PD-L1 expression in gastric cancer cells. Here, we further investigated its influence on T-cell function and activity. Crystal violet staining and absorbance assays were conducted to assess T-cell tumor-killing efficacy. Co-culture of T cells with gastric cancer cells overexpressing CagA (Fig. 6A) and SQLE (Fig. 6B) demonstrated a decrease in T-cell tumor-killing function compared to the control group. These findings indicate that overexpression of CagA and SQLE compromises T-cell-mediated tumor-killing activity.

Additionally, we evaluated T-cell activity and exhaustion. High-resolution live-cell imaging revealed inhibition of Jurkat T cell activity when co-cultured with AGS cells overexpressing CagA and SQLE while there were no significant changes in the activity of AGS cells (Fig. 6C, D). Moreover, co-culture with AGS cells overexpressing CagA and SQLE resulted in increased expression of T cell exhaustion markers, including LAG3, TIM3, and CTLA-4 (Fig. 6E). Thus, it can be inferred that CagA and SQLE impact T-cell activity and function.

### CagA and SQLE promote gastric cancer proliferation and attenuate T cell-mediated tumor killing

**Fig. 7:**
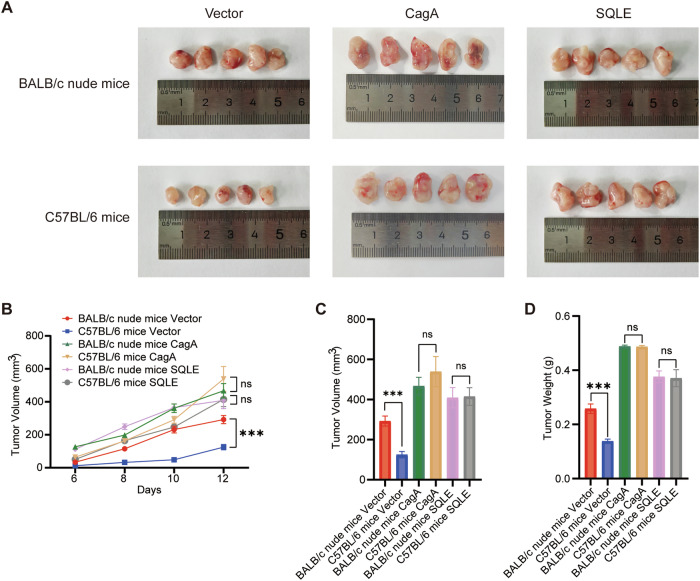
CagA and SQLE promote gastric cancer proliferation and mediate T-cell tumor-killing activity decline. **A** BALB/c nude mice and C57BL/6 mice were subcutaneously injected with equal amounts of stably transfected MFC cells (*n* = 5). **B** Statistical plot of mice tumor growth curve was shown. **C** The tumor volume of BALB/c nude mice and C57BL/6 mice was measured. **D** The tumor weight of BALB/c nude mice and C57BL/6 mice was measured. Data are presented as mean ± SD. **** *P* < 0.0001; *** *P* < 0.001; ** *P* < 0.01; * *P* < 0.05; ns *P* > 0.05.

To further validate the impact of CagA and SQLE on gastric cancer progression, equal numbers of MFC cells were injected into both BALB/c nude mice and C57BL/6 mice. Tumors overexpressing CagA and SQLE exhibited significantly accelerated proliferation compared to the vector control group (Fig. 7A). After inoculation, the tumor volume was regularly measured (Fig. 7B). Interestingly, control cells exhibited a higher proliferation rate in BALB/c nude mice than in C57BL/6 mice due to the immune system’s inhibitory effect. However, upon CagA and SQLE were overexpressed, no significant difference in tumor volume (Fig. 7C) and weight (Fig. 7D) was observed between BALB/c nude mice and C57BL/6 mice. This suggests that CagA and SQLE mitigate the cytotoxic effect of T cells on gastric cancer cells in vivo, promoting immune evasion in gastric cancer. Overall, these findings underscore the pivotal roles of the CagA-SQLE-PD-L1 axis in gastric cancer progression and immune evasion, highlighting their potential roles as therapeutic targets in gastric cancer treatment.

## Discussion

*H. pylori* infection significantly contributes to the onset and progression of gastric cancer. CagA, a primary virulence factor of *H. pylori*, can be injected into gastric epithelial cells via the type IV secretion system [30]. Once internalized, CagA triggers signaling pathways such as PLCγ-MAPK, PI3K-Akt, and Wnt/β-catenin, leading to cellular mutations, epigenetic alterations, and epithelial-mesenchymal transition [31]. However, current research predominantly focuses on *H. pylori*’s impact on intracellular signaling pathways, neglecting its influence on gastric cancer cell metabolism. A recent study has shown that *H. pylori* CagA enhance ether lipid synthesis, thereby increasing lipid peroxidation and susceptibility to ferroptosis in gastric cancer cells. In our study, we compared the transcriptome data of gastric cancer cell lines overexpressing CagA with those of the control cells, revealing differential gene expression enrichment in the sterol biosynthesis pathway. Further investigation uncovered that CagA transcriptionally upregulates SQLE, a crucial enzyme in cholesterol metabolism, leading to elevated cholesterol levels in gastric cancer cells. These findings suggest CagA-positive *H. pylori* infection’s involvement in metabolic reprogramming of gastric cancer.

SQLE exerts a tumorigenic effect in most tumors, with few exceptions. In colorectal cancer, SQLE aids tumor progression by regulating the cell cycle, inhibiting apoptosis, and inducing dysbiosis of the intestinal microbiota [16]. Conversely, it impedes epithelial-mesenchymal transition, thereby attenuating metastasis [17]. In pancreatic cancer, hepatocellular carcinoma, and prostate cancer, SQLE drives tumorigenesis through mechanisms such as activating the PI3K and TGF-β pathways, stimulating cholesterol ester synthesis, and upregulating NADP^+^ [14, 15, 18]. However, SQLE exhibits inhibitory effects on tumor invasion and migration in glioblastoma [32]. Terbinafine, an SQLE inhibitor approved for antifungal therapy, is being investigated for its potential in cancer treatment, showing promising anti-tumor efficacy [18, 33]. Our study identified elevated SQLE expression in CagA-positive gastric cancer. We also investigated the mechanisms underlying CagA-induced transcriptional upregulation of SQLE, implicating YAP1 in this process. Furthermore, our findings clarified the crucial pro-carcinogenic role of SQLE in gastric cancer progression, promoting tumor proliferation, migration, and immune evasion. Whether Terbinafine can be used for the treatment of CagA-positive gastric cancer warrants further investigation.

Activation of PD-1/PD-L1 signaling negatively regulates T cell-mediated immune responses [34]. Despite the approval of several PD-1/PD-L1 monoclonal antibodies for the treatment of various cancers, only 20% to 40% proportion of patients exhibit responsiveness, with fewer achieving long-term remission. Notably, the response rates have not substantially improved, even among patients with PD-L1-positive tumors [35]. Existing antibody-based therapies function by obstructing the conformation of PD-L1 on the cell surface. However, recent research indicates that PD-L1 undergoes palmitoylation modifications and can be sequestered within recycling endosomes, potentially returning to the cell membrane [29, 36]. Consequently, investigating the post-translational modifications and stability of PD-L1 holds significance for a more comprehensive understanding of immune evasion mechanisms in CagA-positive cells.

Various Coenzyme A derivatives are generated during cholesterol synthesis and metabolism. Our study revealed elevated levels of palmitoyl-CoA, a crucial substrate for palmitoylation, in SQLE-expressing cells. SQLE facilitates the production of Pal-CoA, thereby promoting PD-L1 palmitoylation. Further analysis suggests that *H. pylori* CagA impacts PD-L1 post-translational modifications through SQLE, enhancing PD-L1 palmitoylation while reducing ubiquitination. These concurrent actions inhibit PD-L1 degradation via the proteasomal pathway, thus stabilizing its expression. However, this study did not further investigate the sources of Pal-CoA in cells with high SQLE expression, nor did it examine the effects of elevated Pal-CoA levels on the palmitoylation and stability of other downstream proteins. These aspects will be addressed in future research.

Our results align with previous studies indicating that cholesterol stabilizes PD-L1 levels by inhibiting its ubiquitination [22]. We found that the regulation of PD-L1 by SQLE is not solely dependent on cholesterol, as SQLE also increased PD-L1 expression when treated with the cholesterol depletion reagent MCD. Moreover, some studies have suggested a link between cholesterol and protein palmitoylation. Membrane cholesterol is crucial for regulating the influx of exogenous palmitic acid into the cell, as palmitic acid serves as a precursor for Pal-CoA [37]. Cholesterol binds to the LS3 segment in the linker region of Fzd5, facilitating its palmitoylation at the C538 site in pancreatic cancer cells [38]. However, our findings indicate that cholesterol has only a minor effect on PD-L1 palmitoylation in gastric cancer. This does not exclude the possibility of other cholesterol-dependent regulations in gastric cancer, which require further investigation.

Our findings indicate that PD-L1 undergoes intracellular stabilization and, when necessary, can be transported to the cell membrane, thereby assisting CagA-positive gastric cancer cells in evading T cell immune surveillance. This sheds light on how *H. pylori* infection compromises the efficacy of PD-L1-targeted immune therapy in gastric cancer.

## Conclusions

Our investigation elucidated that *H. pylori* CagA induces an upregulation of SQLE, promoting the progression of gastric cancer, as well as leading to an augmentation of PD-L1 palmitoylation and a decrease in ubiquitination, consequently inhibiting T cell activity and facilitating immune evasion. These findings offer new insights into the post-translational regulation of PD-L1 by CagA and uncover novel mechanisms of immune evasion within gastric cancer. Consequently, our study provides insights into innovative therapeutic strategies targeting CagA-positive gastric cancer.

## Supplementary information

Supplementary figures

SUPPLEMENTAL TABLE 1

SUPPLEMENTAL TABLE 2

Original WB data

## Data Availability

The datasets used and/or analysed during the current study are available from the corresponding author on reasonable request.
