# Supplementary material for: Helicobacter pylori CagA promotes gastric cancer immune escape by upregulating SQLE
Source: Cell Death Dis. 2025 Jan 14;16(1):17. doi: 10.1038/s41419-024-07318-w (PMC11733131; doi:10.1038/s41419-024-07318-w)
Supplement: Supplementary file 1 — Supplementary figures [file 41419_2024_7318_MOESM1_ESM.docx]

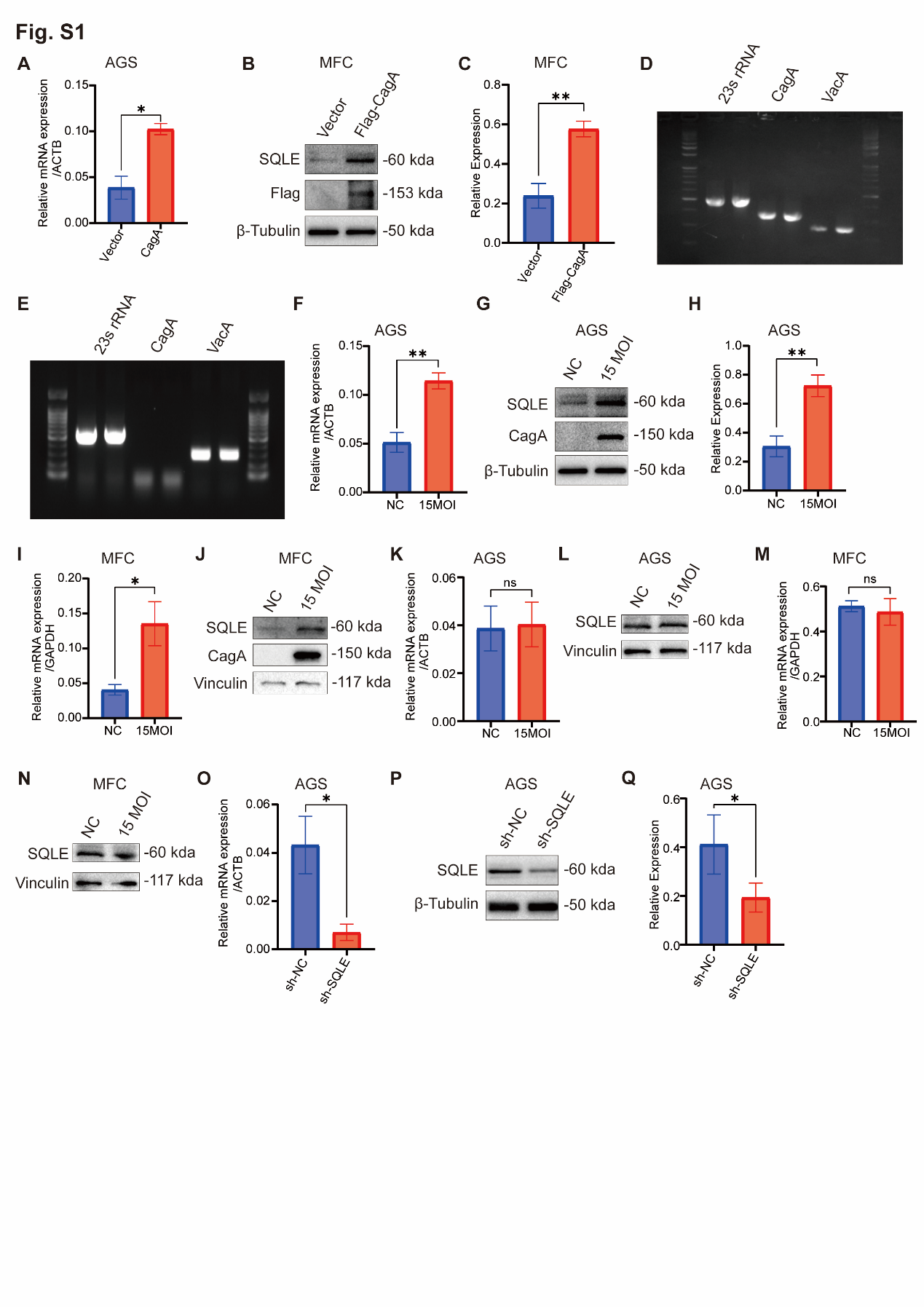


## Fig. S1 *H. pylori* CagA enhances SQLE expression in gastric cancer cells. Related to Fig. 1

**A** qPCR was performed to detect SQLE expression after CagA overexpression in AGS cells.

**B** WB was used to examine SQLE expression after Flag-CagA overexpression in MFC cells, using β-Tubulin as an internal control.

**C** The intensity of SQLE expression (relative to β-Tubulin) in Fig. S1B was quantified with ImageJ (n = 3).

**D-E** Nucleic acid gel electrophoresis was performed to identify *H. pylori* (CagA+) (D) and *H. pylori* (CagA-) (E) strains.

**F** qPCR was used to detect SQLE expression in AGS cells co-cultured with the *H. pylori* (CagA+) strain.

**G** WB was used to detect SQLE expression in AGS cells co-cultured with the *H. pylori* (CagA+) strain, using β-Tubulin as an internal control.

**H** The intensity of SQLE expression (relative to β-Tubulin) in Fig. S1G was quantified with ImageJ (n = 3).

**I** qPCR was used to detect SQLE expression in MFC cells co-cultured with the *H. pylori* (CagA+) strain.

**J** WB was used to detect SQLE expression in MFC cells co-cultured with the *H. pylori* (CagA+) strain, using vinculin as an internal control.

**K** qPCR was used to detect SQLE expression in AGS cells co-cultured with the *H. pylori* (CagA-) strain.

**L** WB was used to detect SQLE expression in AGS cells co-cultured with the *H. pylori* (CagA-) strain, using vinculin as an internal control.

**M** qPCR was used to detect SQLE expression in MFC cells co-cultured with the *H. pylori* (CagA-) strain.

**N** WB was used to detect SQLE expression in MFC cells co-cultured with the *H. pylori* (CagA-) strain, using vinculin as an internal control.

**O** qPCR was used to detect the transfection efficiency of SQLE from the mRNA level after the SQLE knockdown in AGS cells.

**P** WB was used to detect the transfection efficiency of SQLE from the protein level after the SQLE knockdown in AGS cells. β-Tubulin was used as an internal control in WB analysis.

**Q** The intensity of SQLE expression (relative to β-Tubulin) in Fig. S1P was quantified with ImageJ (n = 3).

Data are presented as mean ± SD. **** *P* < 0.0001; *** *P* < 0.001; ** *P* < 0.01; * *P* < 0.05; ns *P* > 0.05.


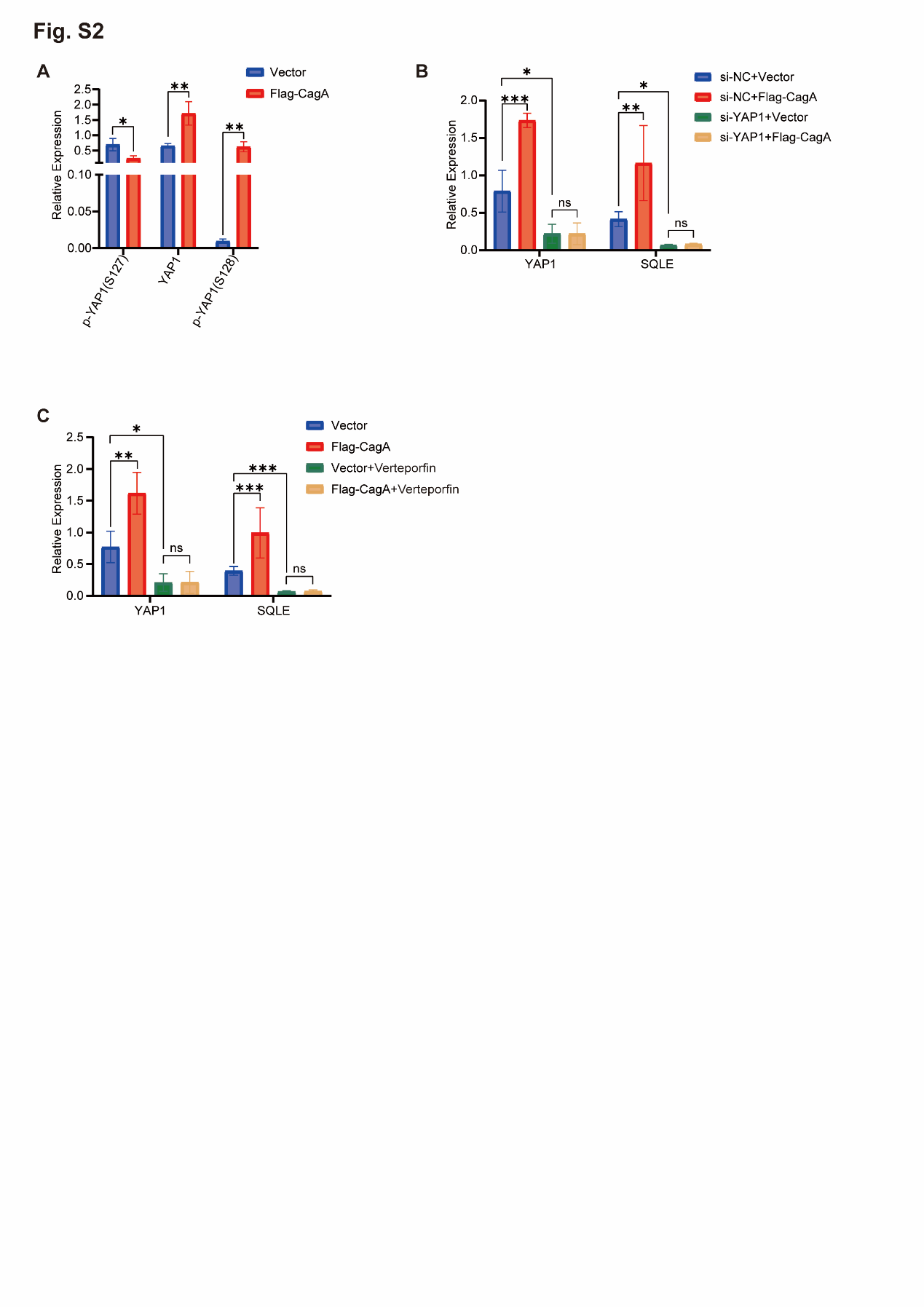


## Fig. S2 *H. pylori* CagA regulates SQLE expression through YAP1. Related to Fig. 2

**A** The intensity of p-YAP1 (S127), YAP1, and p-YAP1 (S128) expression (relative to β-Tubulin) in Fig. 2C was quantified with ImageJ (n = 3).

**B** The intensity of YAP1 and SQLE expression (relative to β-Tubulin) in Fig. 2G was quantified with ImageJ (n = 3).

**C** The intensity of YAP1 and SQLE expression (relative to β-Tubulin) in Fig. 2H was quantified with ImageJ (n = 3).

Data are presented as mean ± SD. **** *P* < 0.0001; *** *P* < 0.001; ** *P* < 0.01; * *P* < 0.05; ns *P* > 0.05.


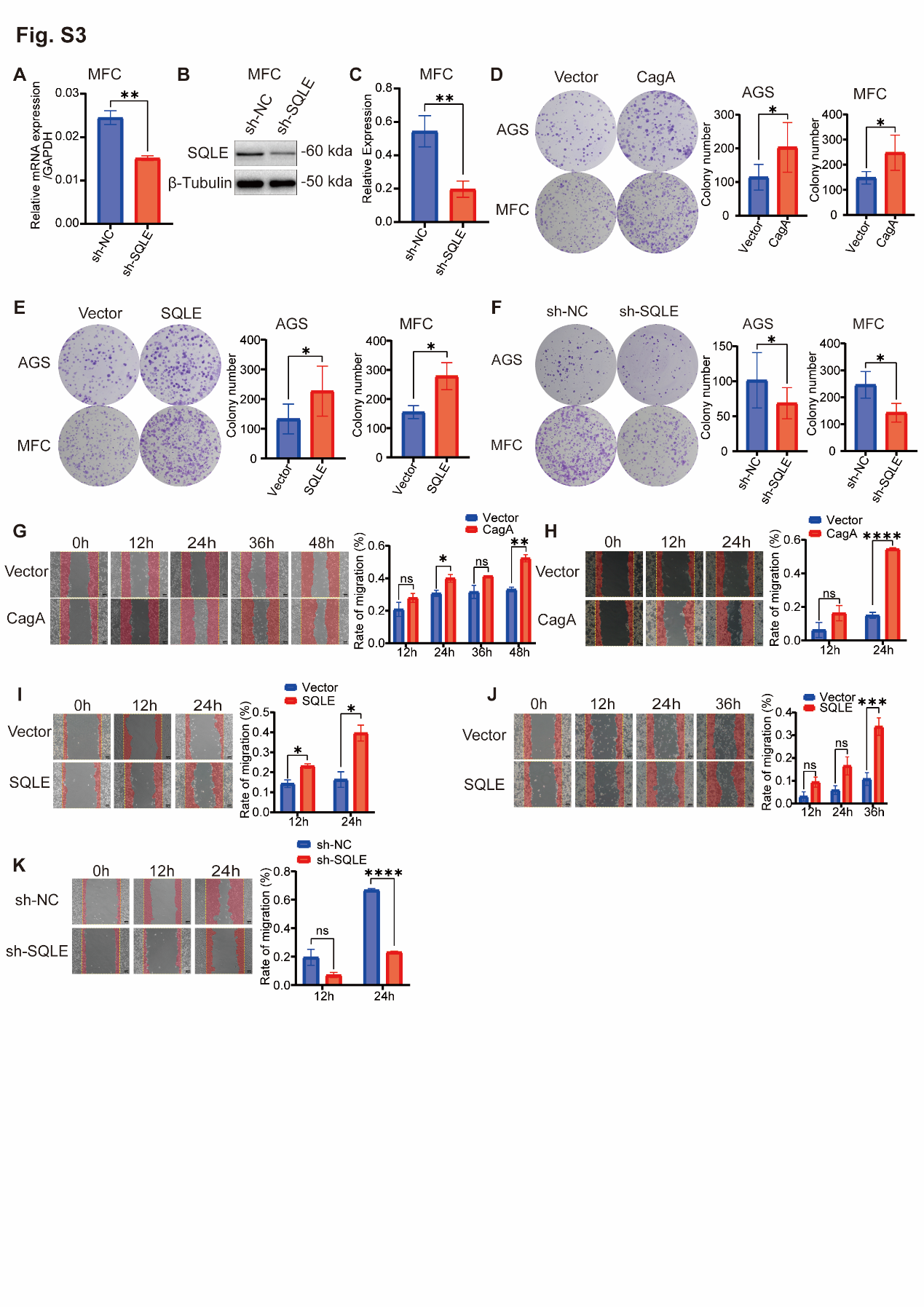


## Fig. S3 CagA and SQLE promote gastric cancer progression. Related to Fig. 3

**A** qPCR was used to detect the transfection efficiency of SQLE from the mRNA level after the SQLE knockdown in MFC cells.

**B** WB was used to detect the transfection efficiency of SQLE from the protein level after the SQLE knockdown in MFC cells, using β-Tubulin as an internal control.

**C** The intensity of SQLE expression (relative to β-Tubulin) in Fig. S3B was quantified with ImageJ (n = 3).

**D** Left, colony formation assays were used to detect the proliferation of AGS and MFC cells after CagA overexpression. Right, quantitative analysis results of it (n = 3).

**E** Left, colony formation assays were used to detect the proliferation of AGS and MFC cells after SQLE overexpression. Right, quantitative analysis results of it (n = 3).

**F** Left, colony formation assays were used to detect the proliferation of AGS and MFC cells after SQLE knockdown. Right, quantitative analysis results of it (n = 3).

**G-H** Left, wound healing assays were used to examine the migration of AGS (G) and MFC (H) cells after CagA overexpression. Right, quantitative analysis results of it (n = 3). Scale bar = 100 µm.

**I-J** Left, wound healing assays were used to examine the migration of AGS (I) and MFC (J) cells after SQLE overexpression. Right, quantitative analysis results of it (n = 3). Scale bar = 100 µm.

**K** Wound healing assays were used to examine the migration of AGS cells after SQLE knockdown. Right, quantitative analysis results of it (n = 3). Scale bar = 100 µm.

Data are presented as mean ± SD. **** *P* < 0.0001; *** *P* < 0.001; ** *P* < 0.01; * *P* < 0.05; ns *P* > 0.05.


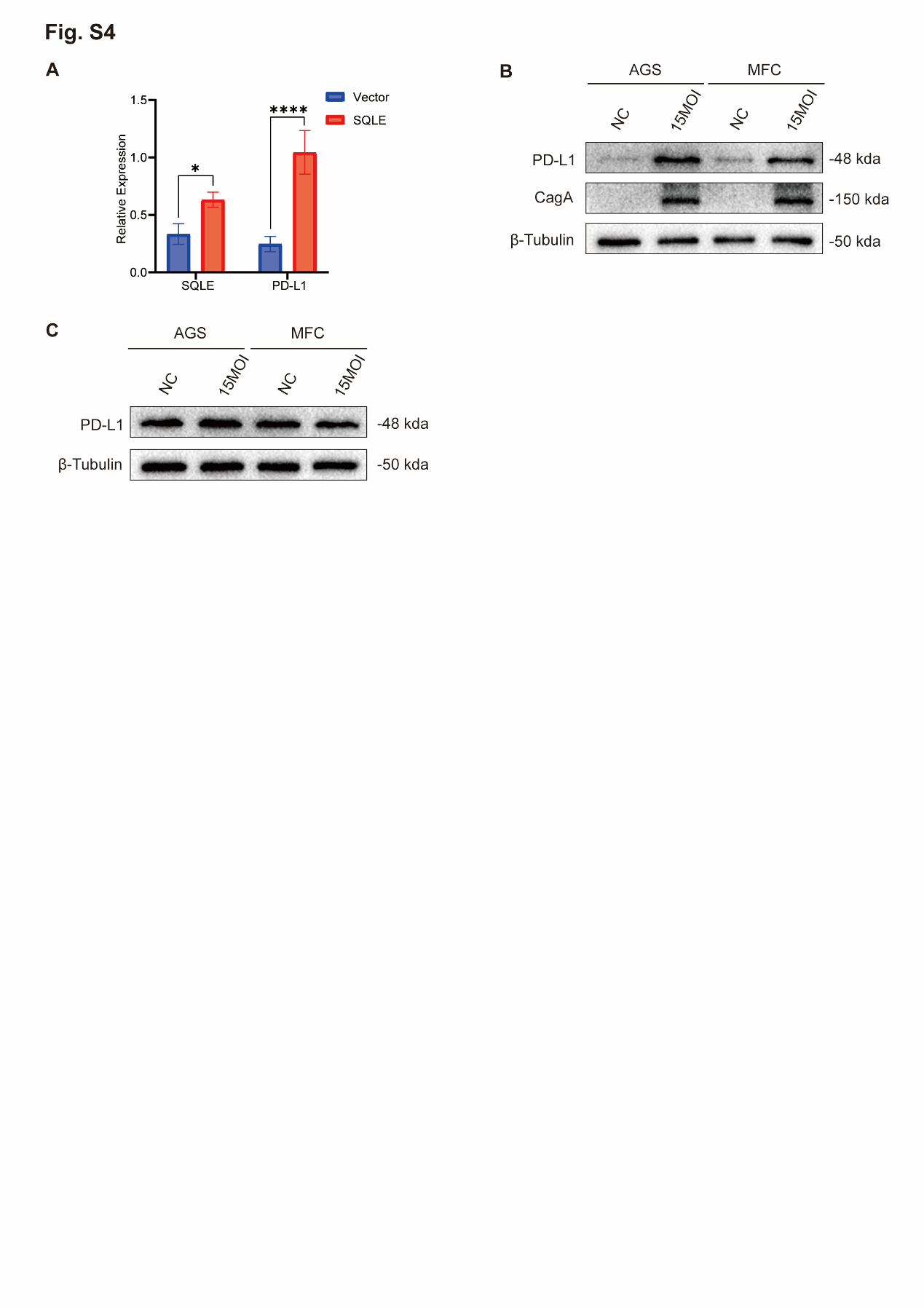


## Fig. S4 *H. pylori* (CagA+) promote PD-L1 expression in gastric cancer cells. Related to Fig. 4

**A** The intensity of SQLE and PD-L1 expression (relative to β-Tubulin) in Fig. 4F was quantified with ImageJ (n = 3).

**B** WB analysis was performed to identify PD-L1 expression after co-culturing with the *H. pylori* (CagA+) strain in AGS and MFC cells, using β-Tubulin as an internal control.

**C** WB analysis was performed to identify PD-L1 expression after co-culturing with the *H. pylori* (CagA-) strain in AGS and MFC cells, using β-Tubulin as an internal control.

Data are presented as mean ± SD. **** *P* < 0.0001; *** *P* < 0.001; ** *P* < 0.01; * *P* < 0.05; ns *P* > 0.05.


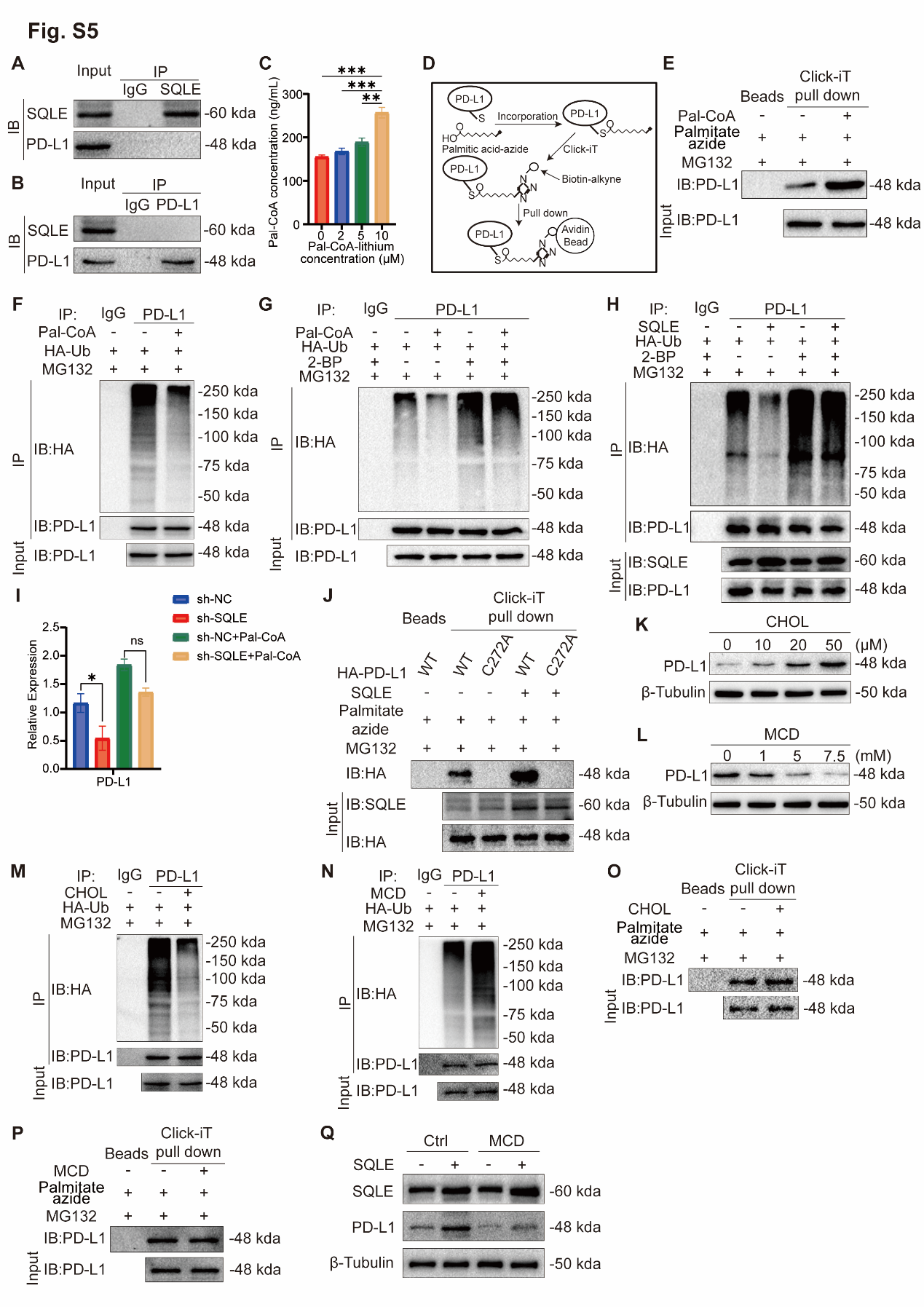


## Fig. S5 SQLE promotes PD-L1 palmitoylation and inhibits its ubiquitination. Related to Fig. 5

**A-B** Co-IP assays were performed to detect the interaction between SQLE and PD-L1.

**C** Elisa assay was performed to detect palmitoyl-CoA (Pal-CoA) concentration.

**D-E** Click-iT reaction (D) was used to detect PD-L1 palmitoylation in AGS cells incubated with Pal-CoA (10 μM for 48 hours) (E). Click-iT palmitate azide (100 μM for 6 hours) and MG132 (10 μM for 6 hours) were added before the samples were collected.

**F** Co-IP was used to detect PD-L1 ubiquitination after MG132 (10 μM for 6 hours) incubation in AGS cells treated with Pal-CoA (10 μM for 48 hours).

**G** Co-IP was used to detect PD-L1 ubiquitination after 2-BP (100 μM for 24 hours) and MG132 (10 μM for 6 hours) incubation in AGS cells treated with Pal-CoA (10 μM for 48 hours).

**H** Co-IP was used to detect PD-L1 ubiquitination after 2-BP (100 μM for 24 hours) and MG132 (10 μM for 6 hours) incubation in MFC cells overexpressing SQLE.

**I** The intensity of PD-L1 expression (relative to β-Tubulin) in Fig. 5J was quantified with ImageJ (n = 3).

**J** Click-iT was used to detect PD-L1 palmitoylation in AGS cells after transfection with HA-PD-L1 (wild type (WT) or C272A) and overexpression of SQLE. Click-iT palmitate azide (100 μM for 6 hours) and MG132 (10 μM for 6 hours) were added before the samples were collected.

**K-L** WB was used to examine the protein level of PD-L1 in AGS cells after the use of cholesterol (CHOL, 0 to 50 μM for 12 hours) (K) and methyl-β-cyclodextrin (MCD, 0 to 7.5 mM for 6 hours) (L). β-Tubulin was used as an internal control.

**M-N** Co-IP was used to detect PD-L1 ubiquitination after MG132 (10 μM for 6 hours) incubation in AGS cells treated with CHOL (50 μM for 12 hours) (M) and MCD (7.5 mM for 6 hours) (N).

**O-P** Click-iT reaction was used to detect PD-L1 palmitoylation in AGS cells treated with CHOL (50 μM for 12 hours) (O) and MCD (7.5 mM for 6 hours) (P). Click-iT palmitate azide (100 μM for 6 hours) and MG132 (10 μM for 6 hours) were added before the samples were collected.

**Q** WB was used to detect PD-L1 protein levels in AGS cells overexpressing SQLE incubated with MCD (7.5 mM for 6 hours), using β-Tubulin as an internal control.

Data are presented as mean ± SD. **** *P* < 0.0001; *** *P* < 0.001; ** *P* < 0.01; * *P* < 0.05; ns *P* > 0.05.
