# Supplementary material for: Helicobacter pylori CagA promotes gastric cancer immune escape by upregulating SQLE
Source: Cell Death Dis. 2025 Jan 14;16(1):17. doi: 10.1038/s41419-024-07318-w (PMC11733131; doi:10.1038/s41419-024-07318-w)
Supplement: Supplementary file 4 — Original WB data [file 41419_2024_7318_MOESM4_ESM.docx]

**Original WB data**

**Figure 1E**

**
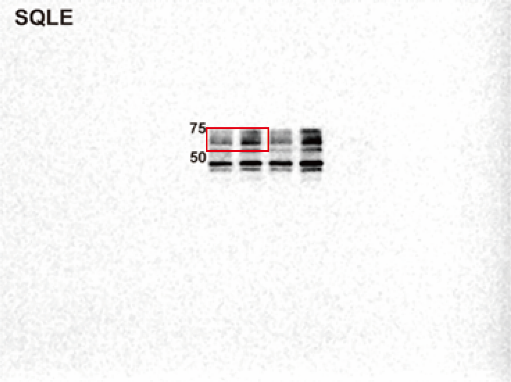

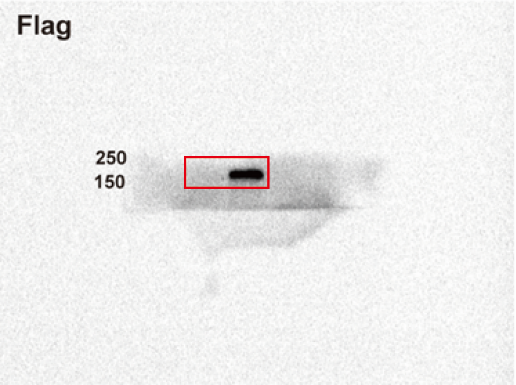
**

**
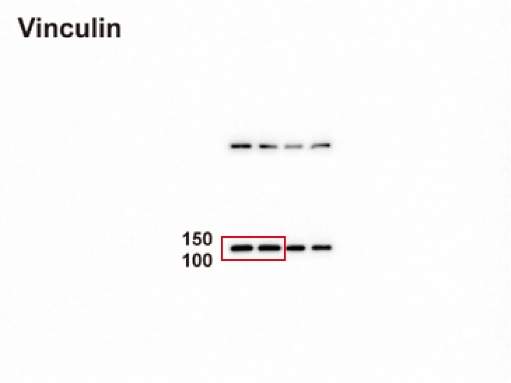
**

**Figure 2C**


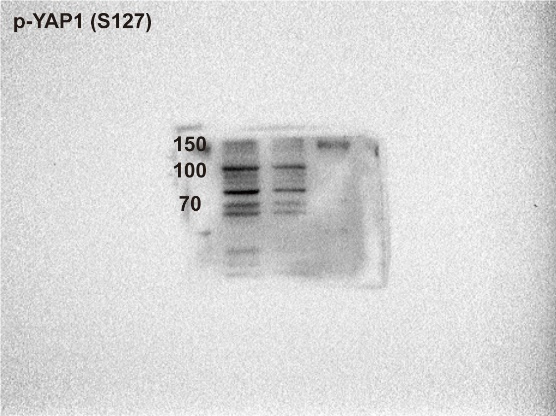

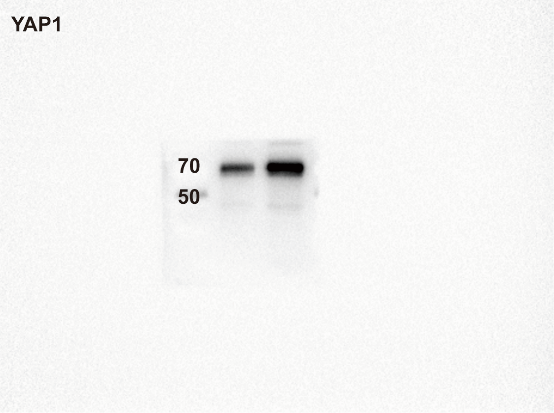


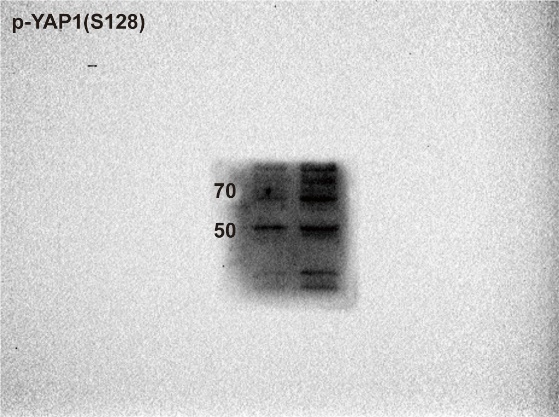

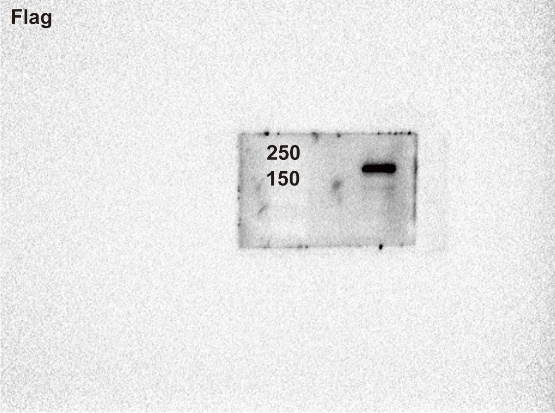


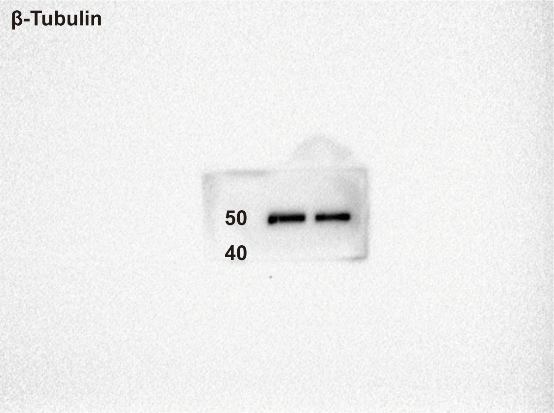


**Figure 2D**


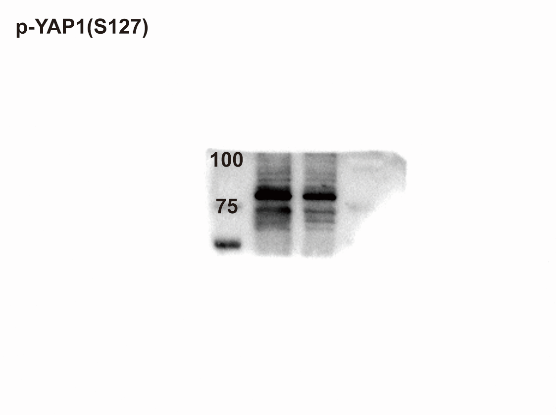

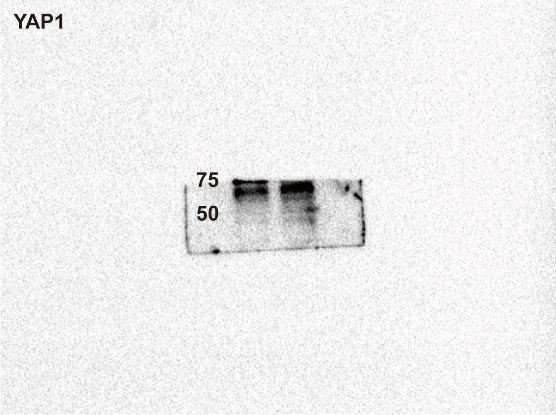


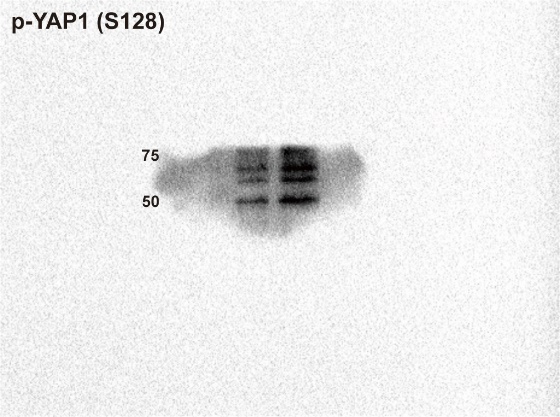

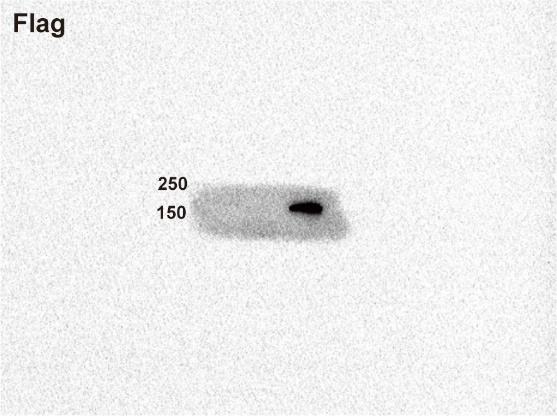


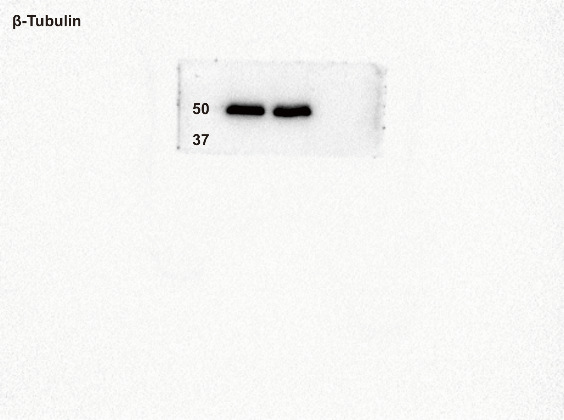


**Figure 2G**


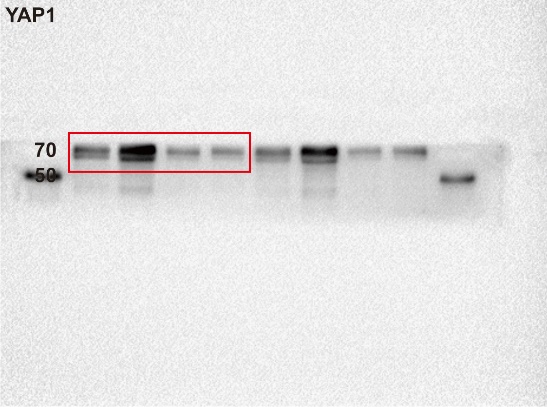

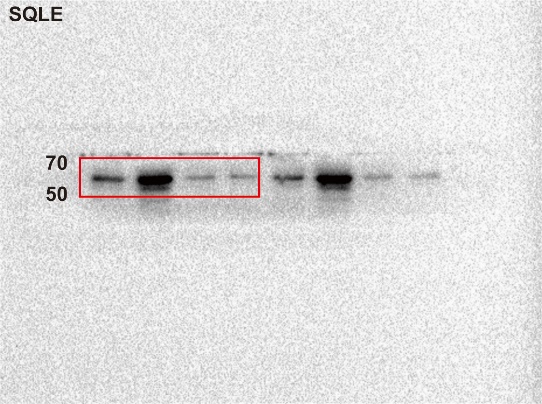


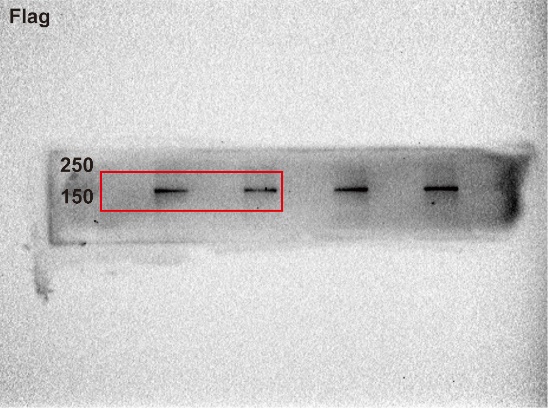

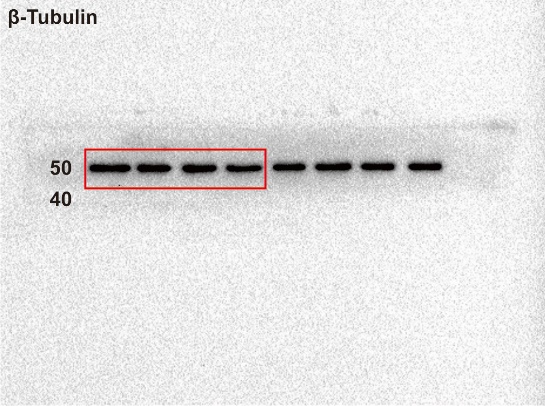


**Figure 2H**


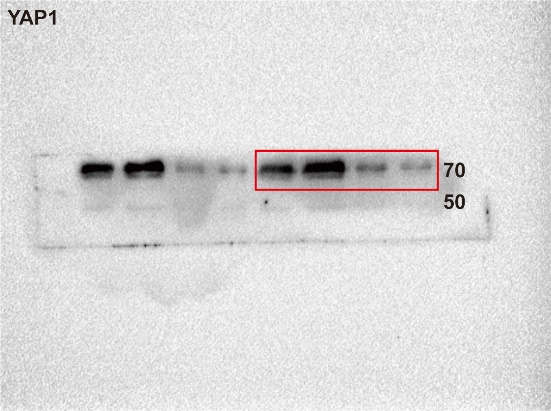

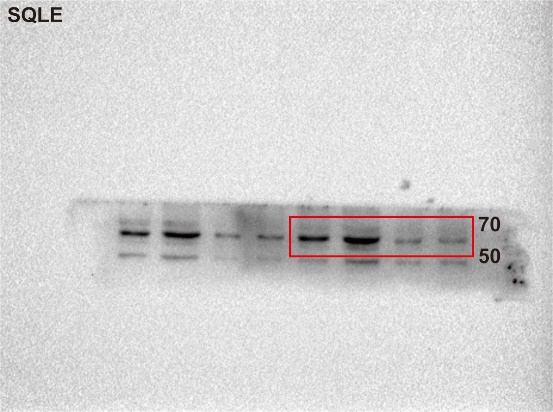


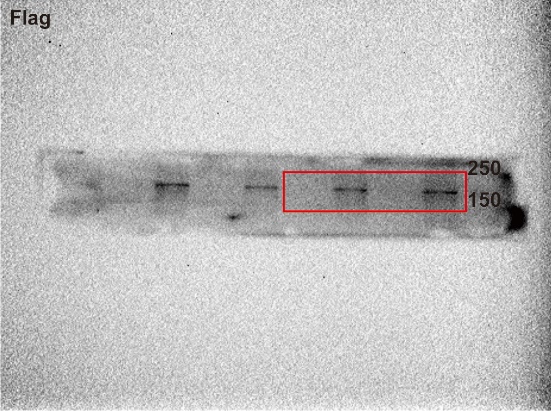

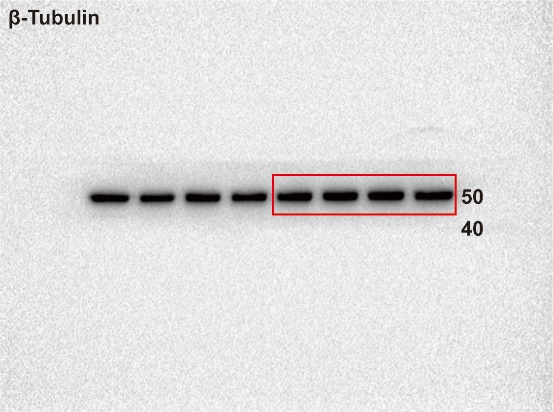


**Figure 4D**

**
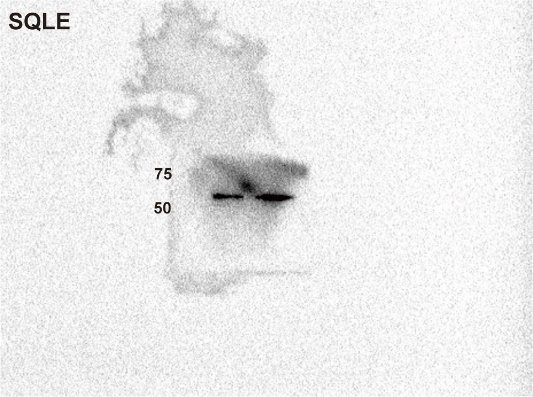
**
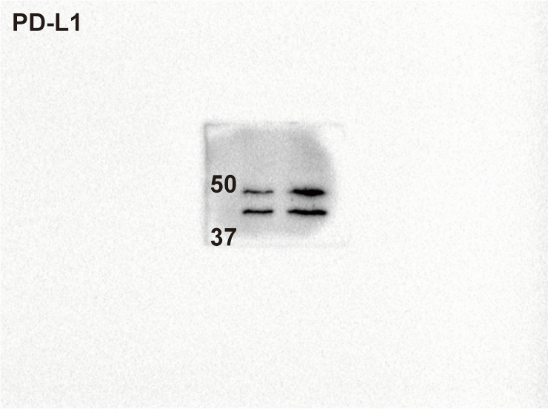


**
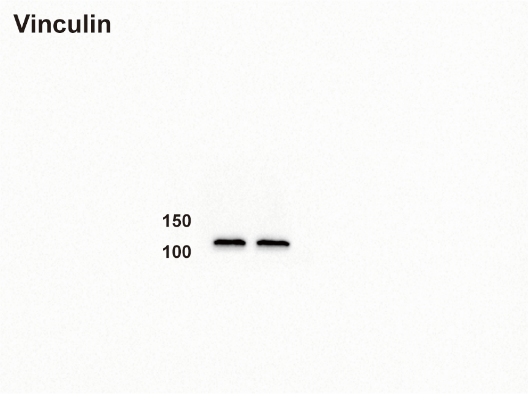
**

**Figure 4E**


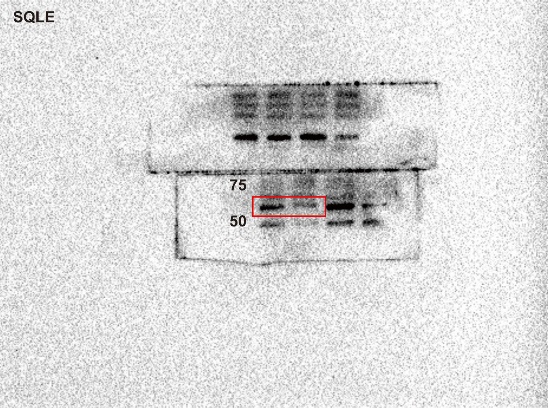

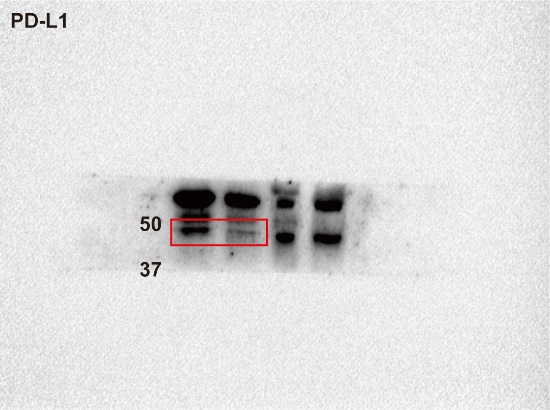


**
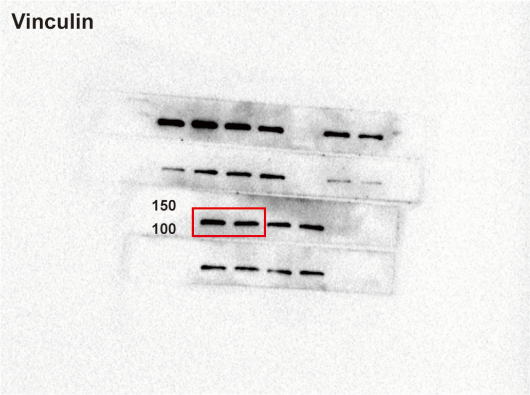
**

**Figure 4F**

**
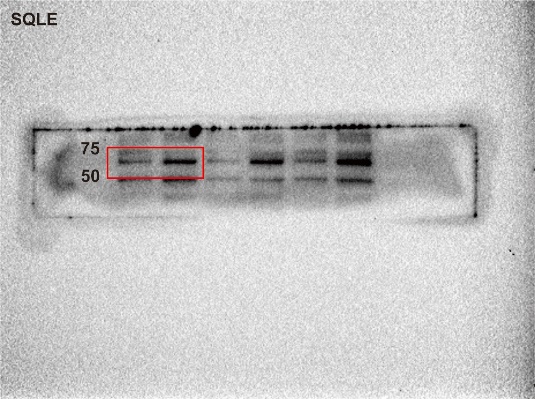

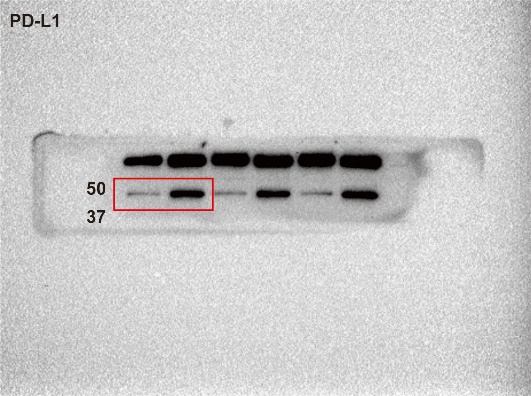
**

**
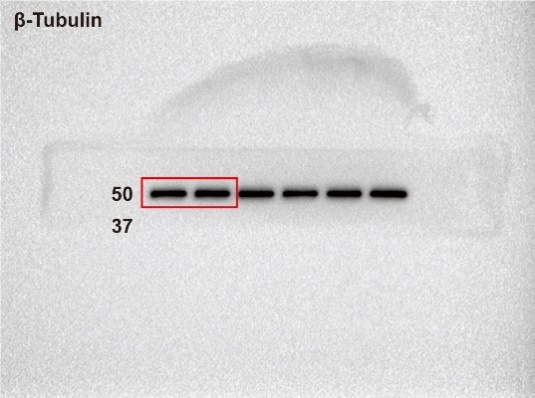
**

**Figure 4G**

**
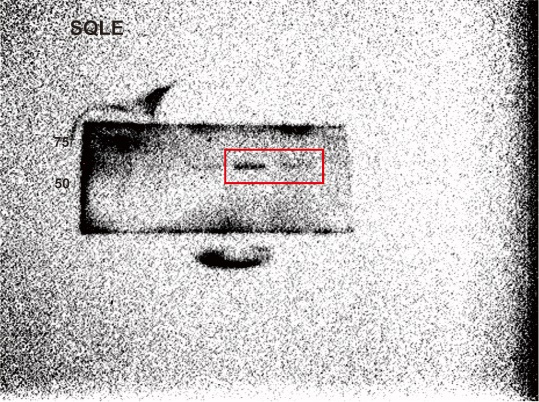

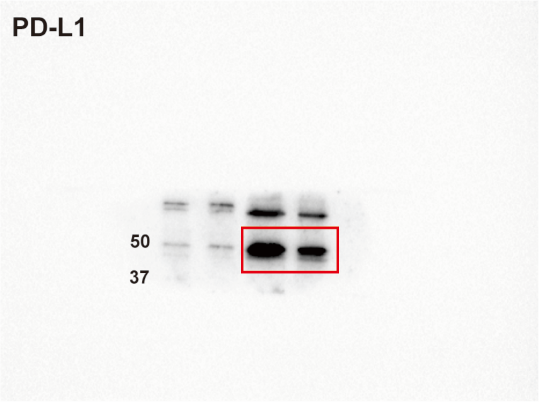
**

**
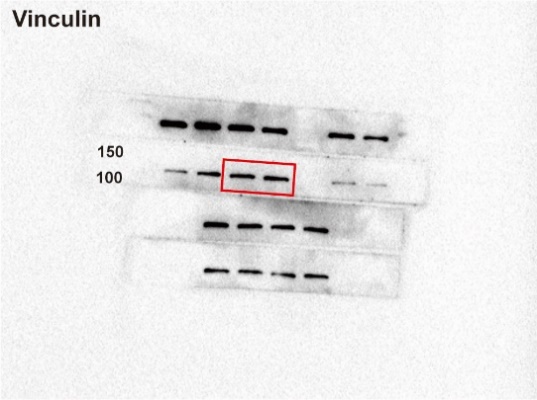
**

**Figure 4H**

**
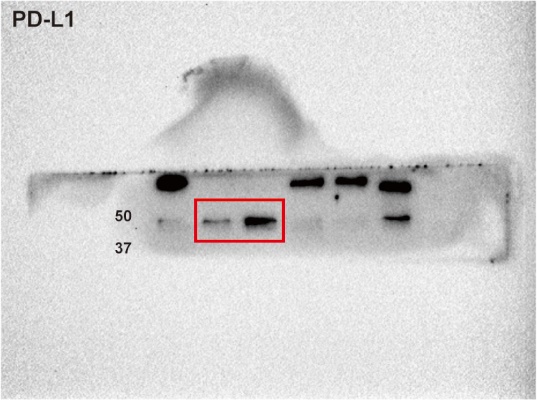

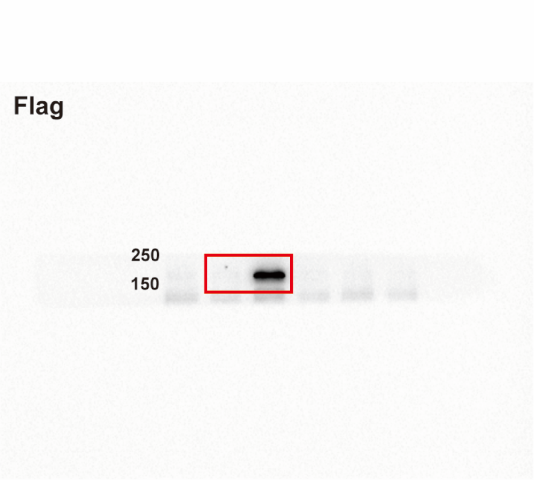
**

**
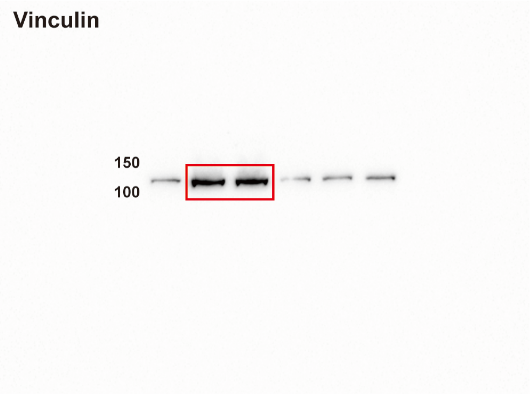
**

**Figure 4I**

**
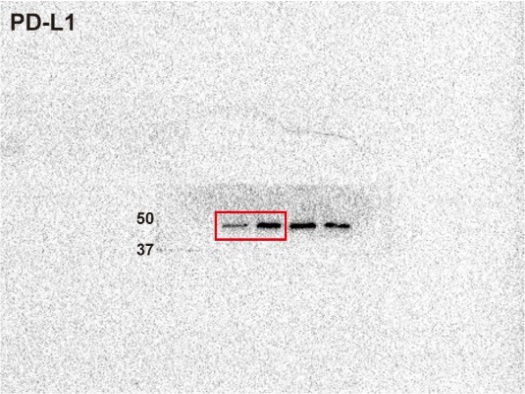

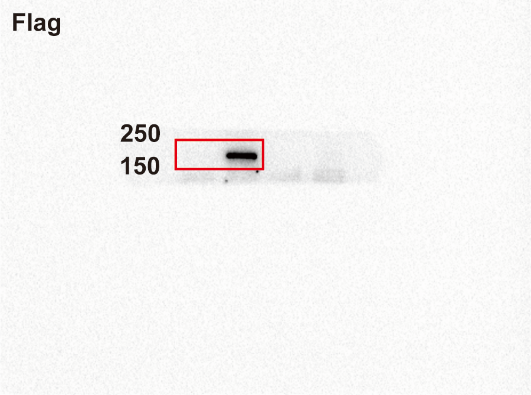
**

**
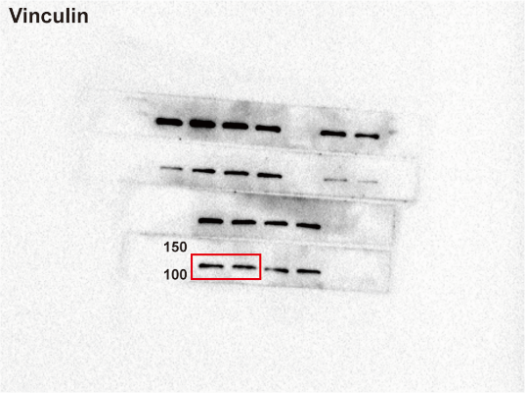
**

**Figure 4J**

**
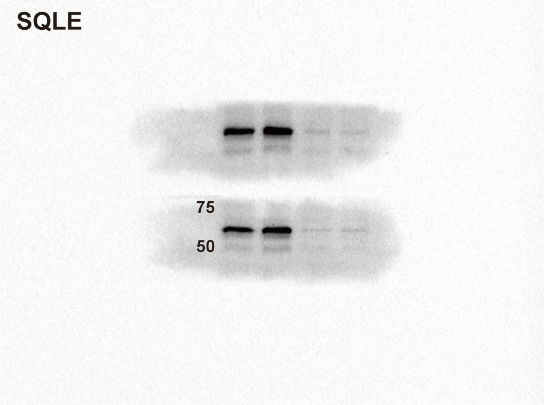

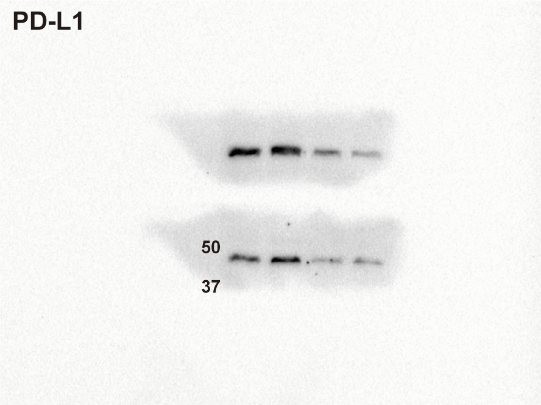
**

**
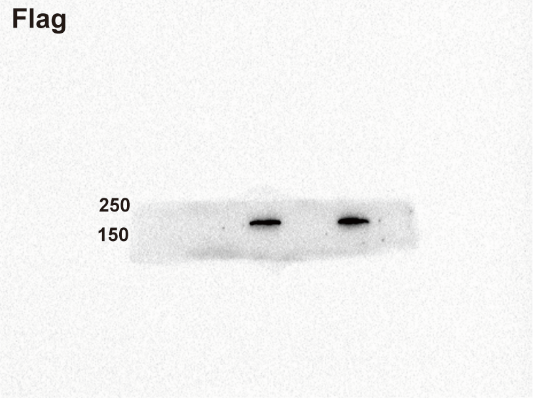

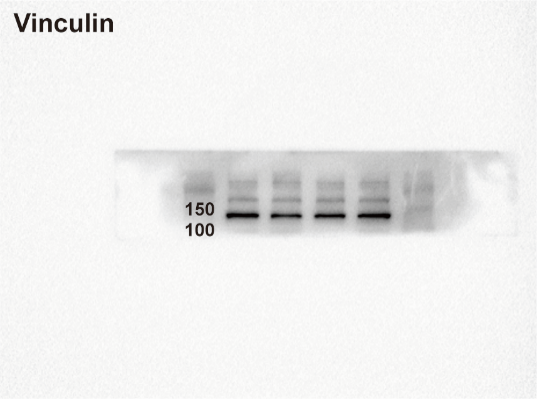
**

**Figure 5A**

**
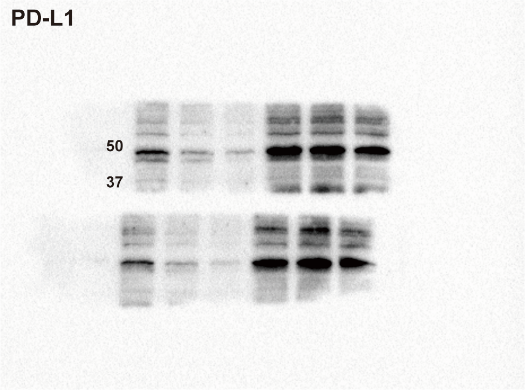

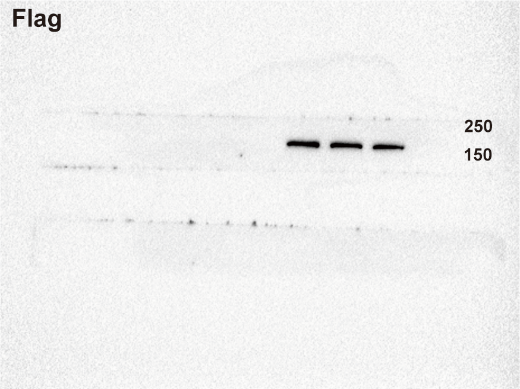
**

**
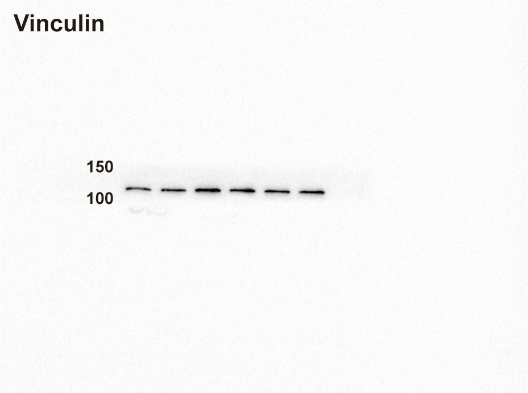
**

**Figure 5B**

**
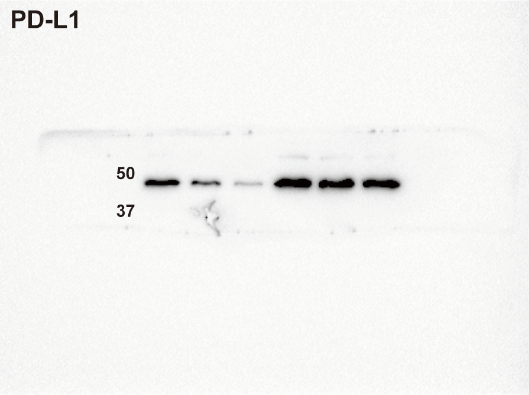

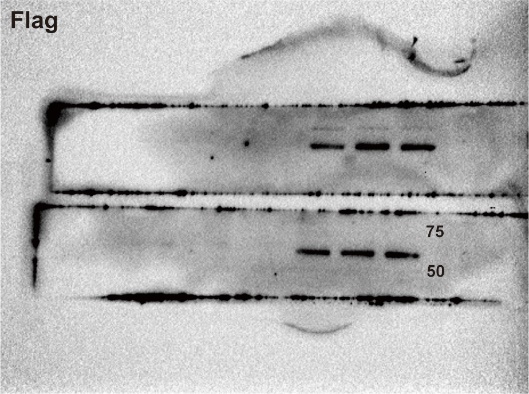
**

**
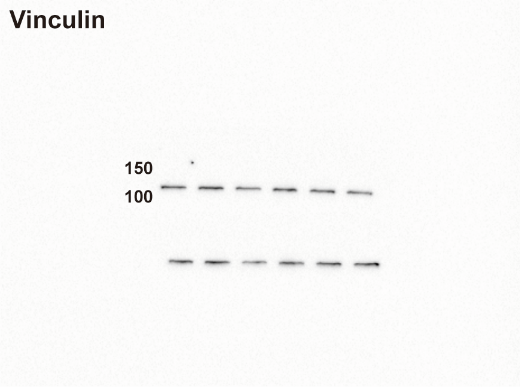
**

**Figure 5C**

**
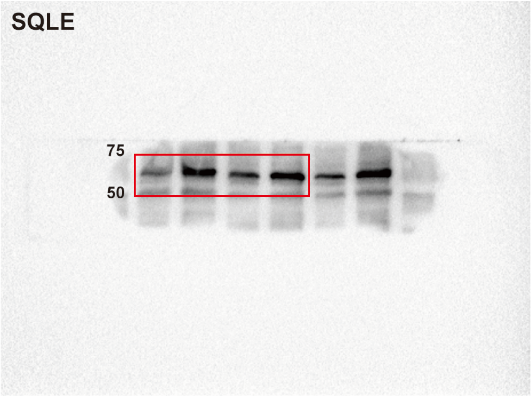

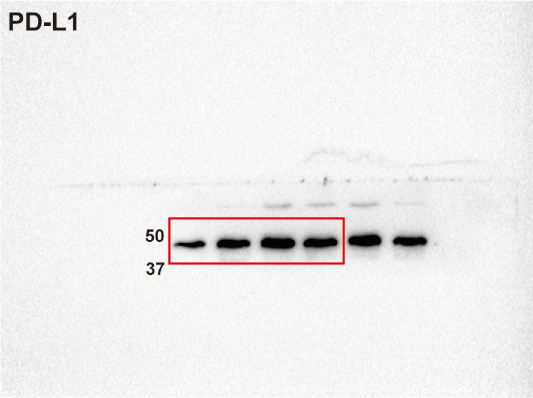
**

**
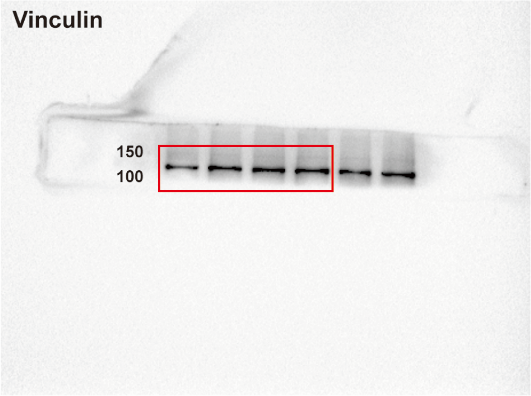
**

**Figure 5D**

**
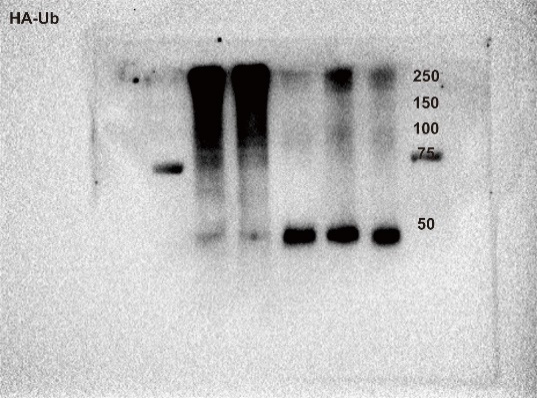

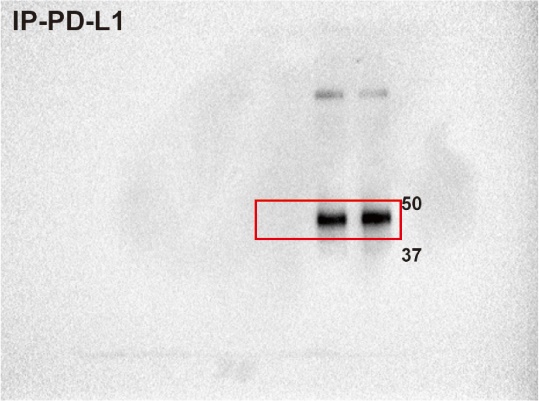
**

**
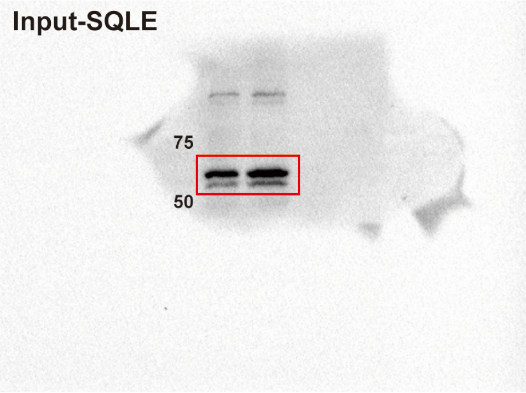
**
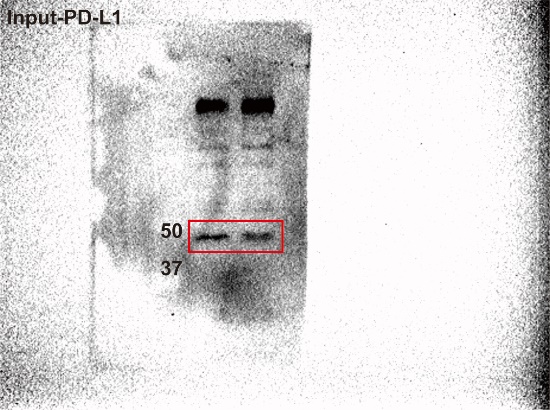


**Figure 5F**

**
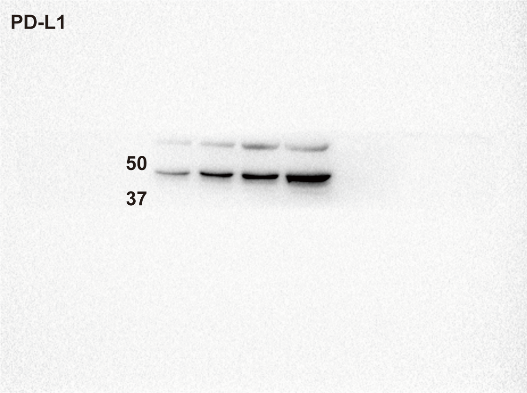

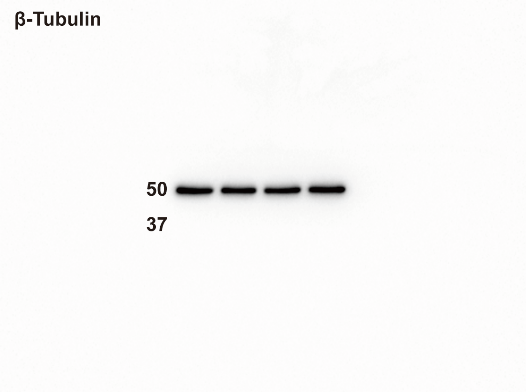
**

**Figure 5G**


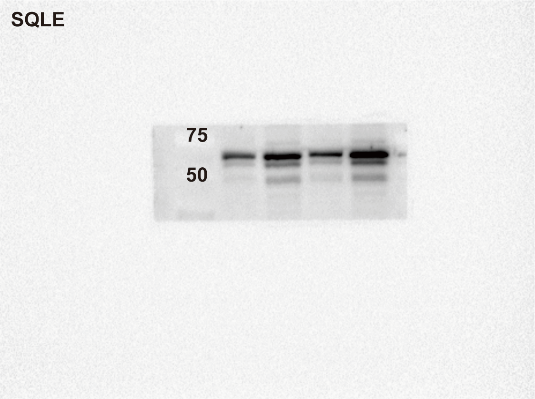

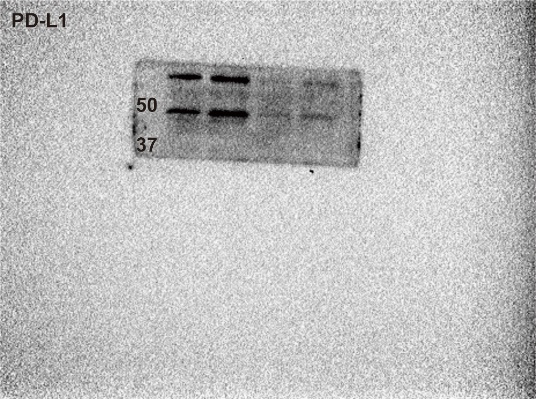


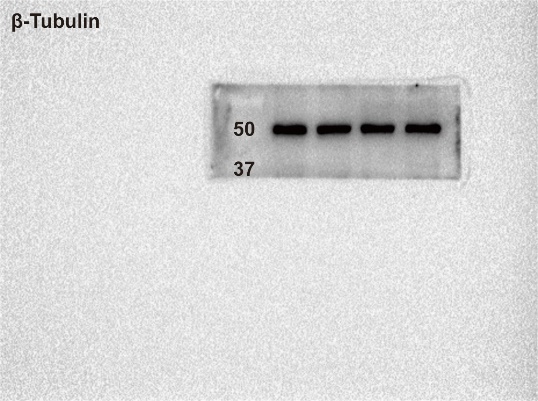


**Figure 5H**

**
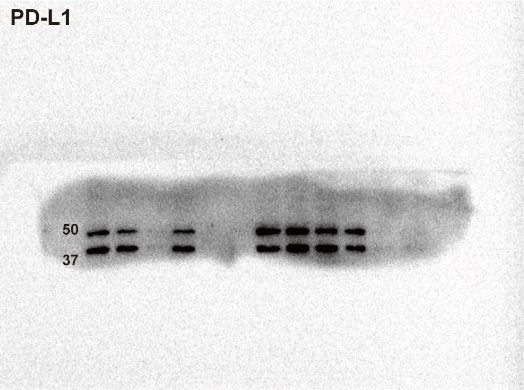

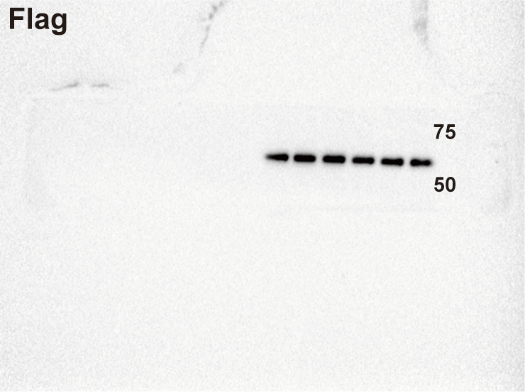
**

**
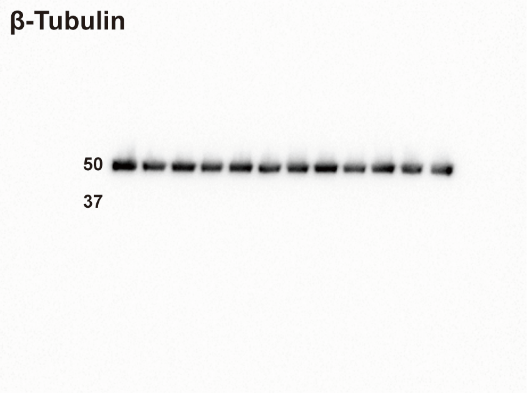
**

**Figure 5I**

**
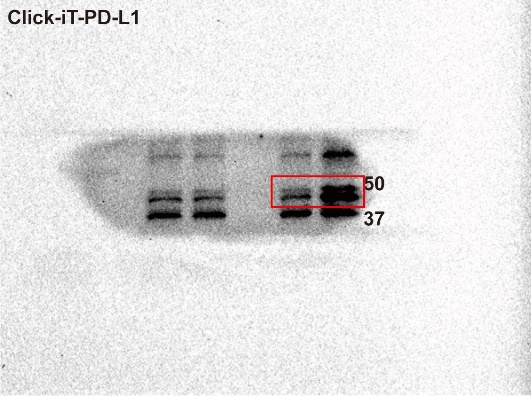

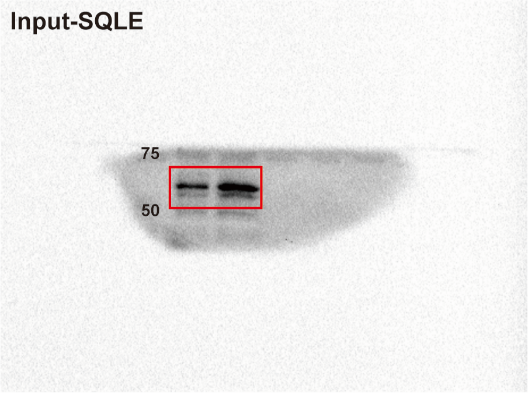
**

**
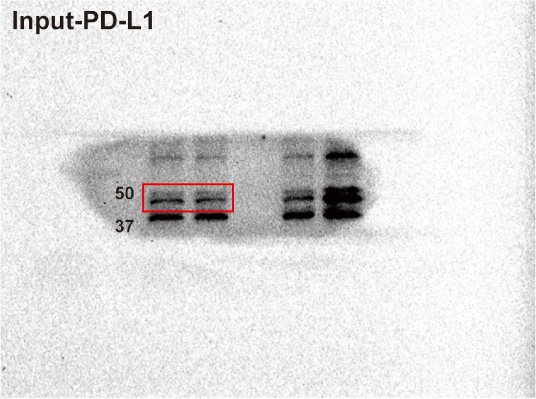
**

**Figure 5J**

**
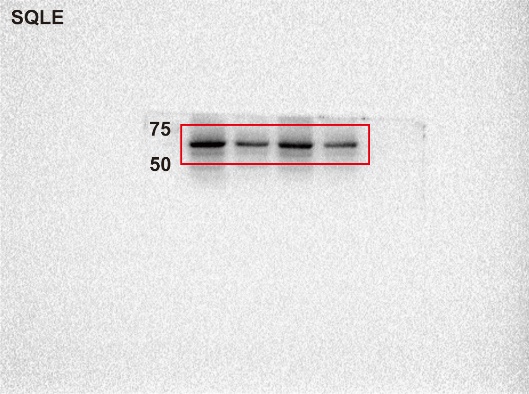

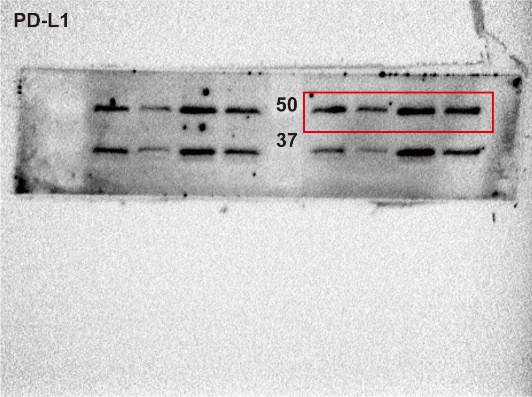
**

**
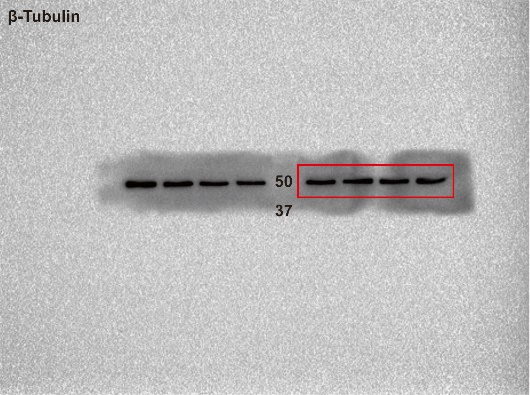
**

**Figure 5K**

**
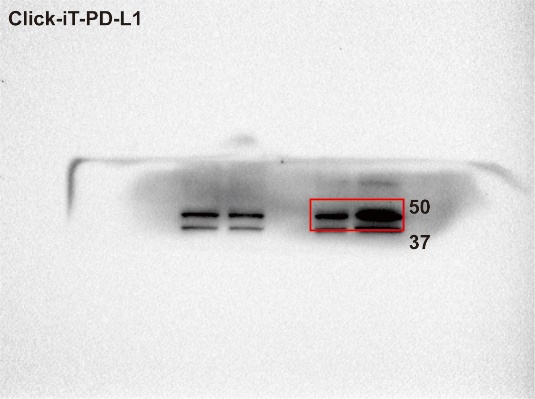

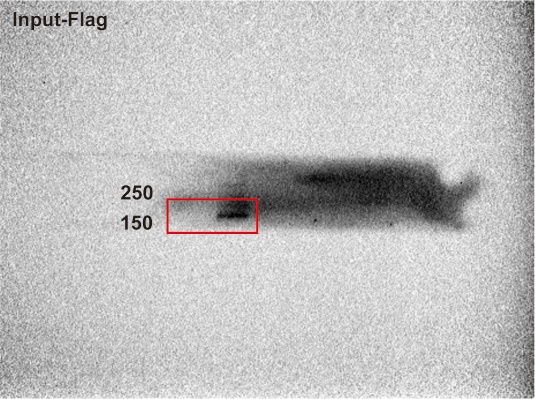
**

**
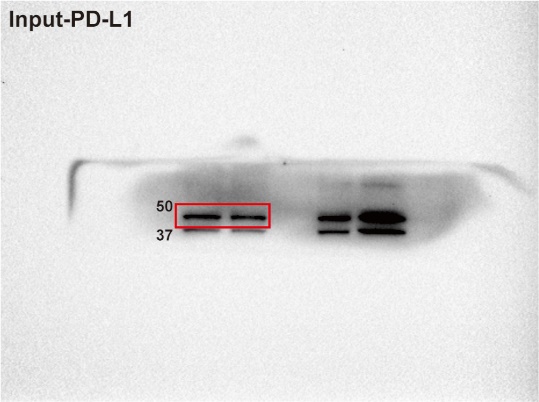
**

**Figure 5L**


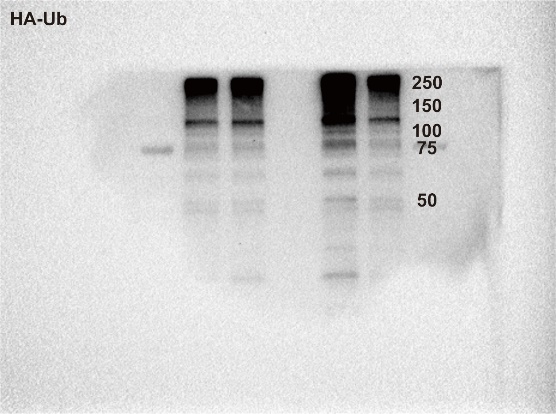
**
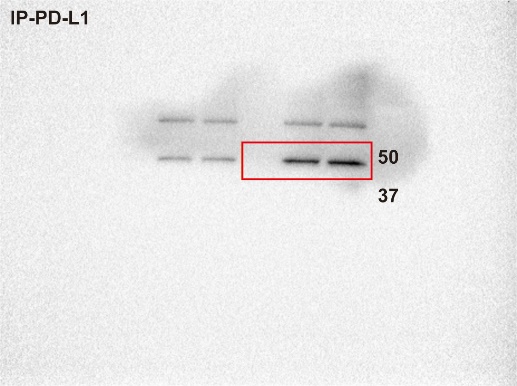
**

**
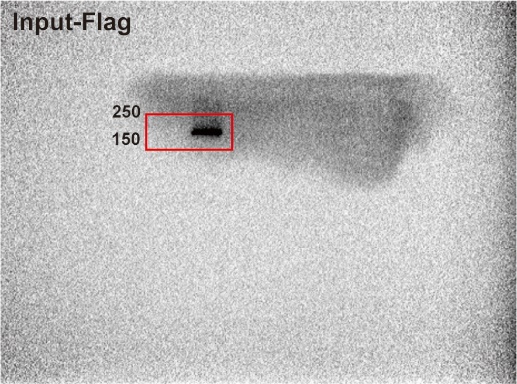

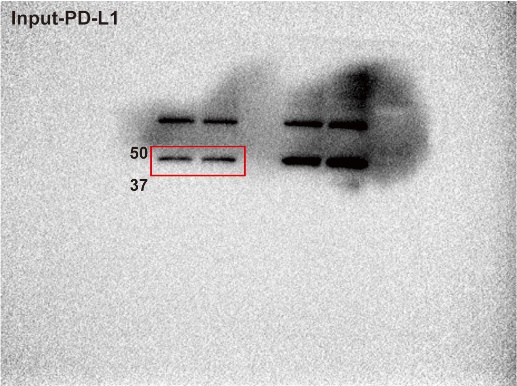
**

**Figure 5M**


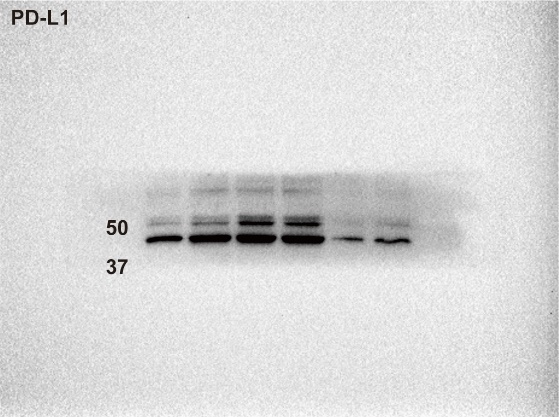
**
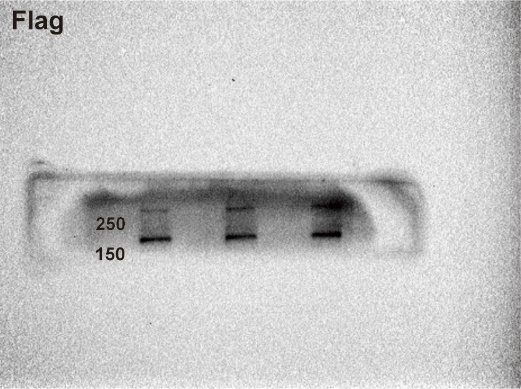
**

**
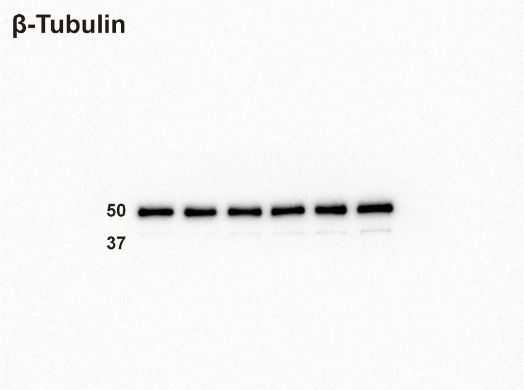
**

**Figure 5N**


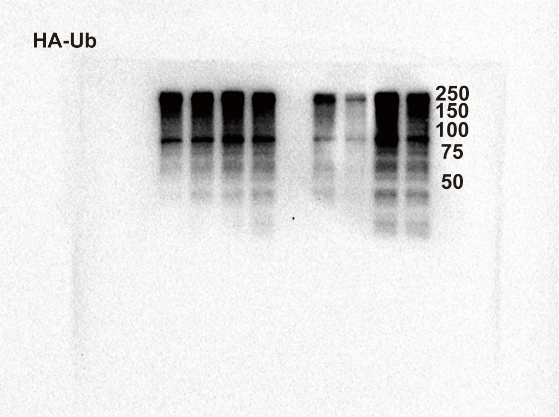
**
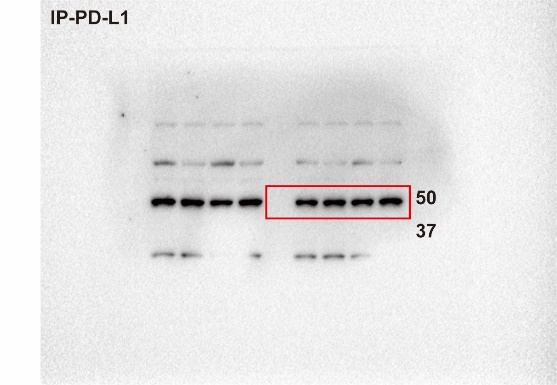
**

**
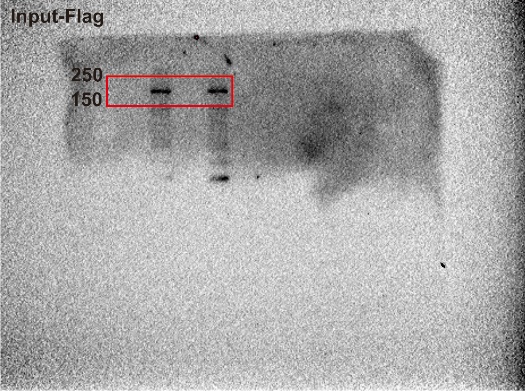

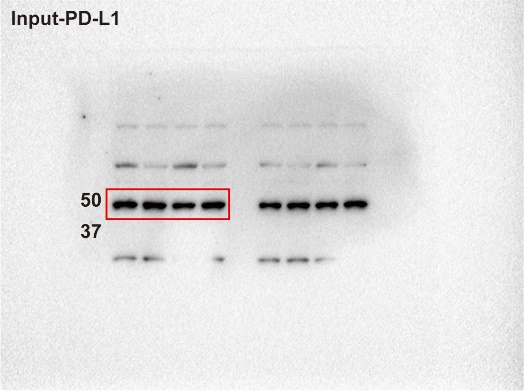
**

**Figure 6E**


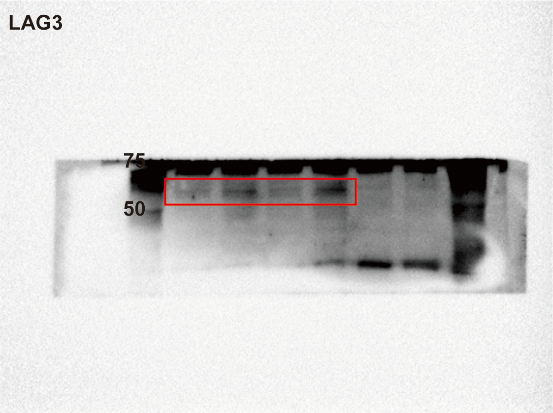
**
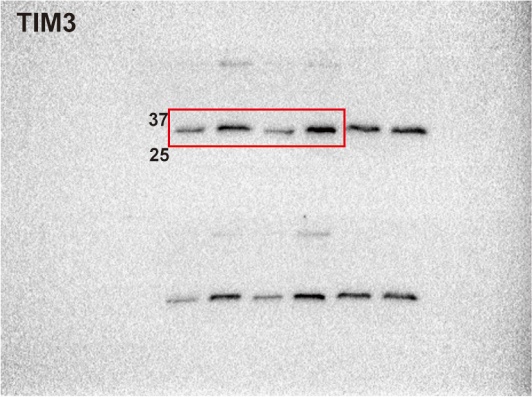
**


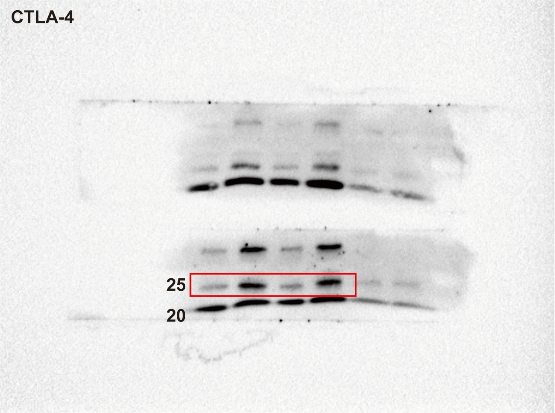
**
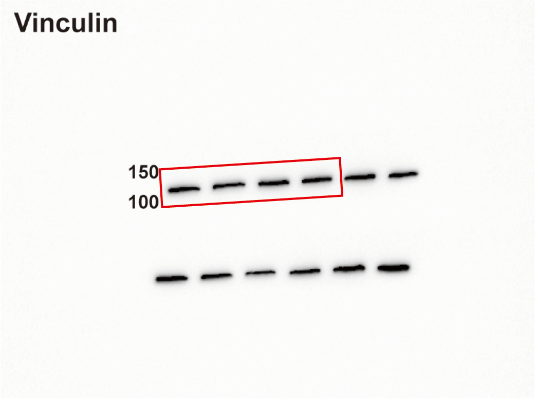
**

**Figure S1B**

**
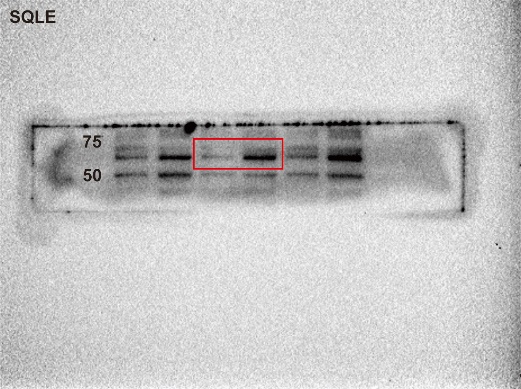

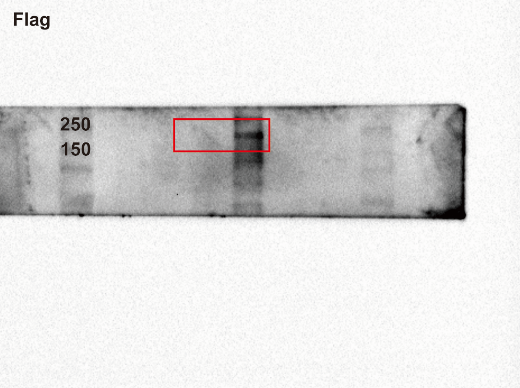
**

**
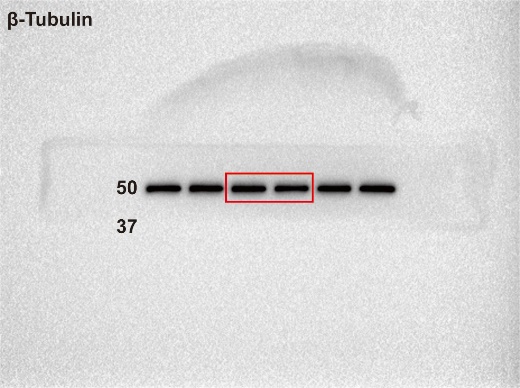
**

**Figure S1G**

**
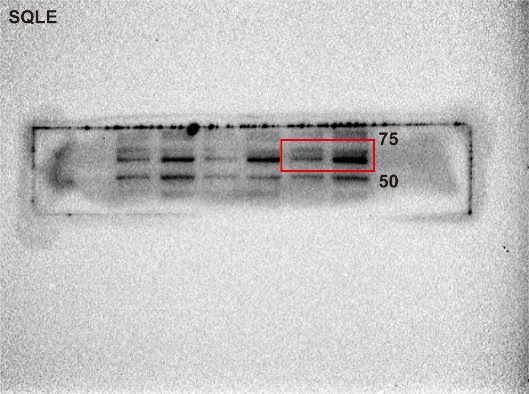

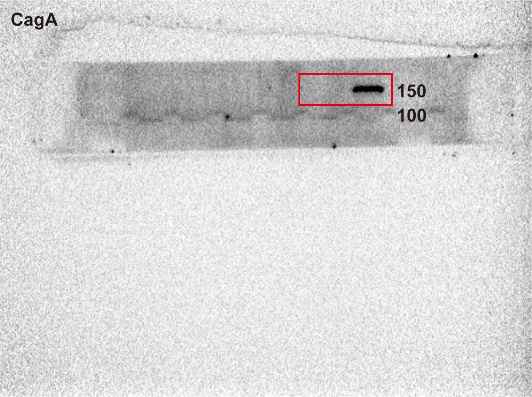
**

**
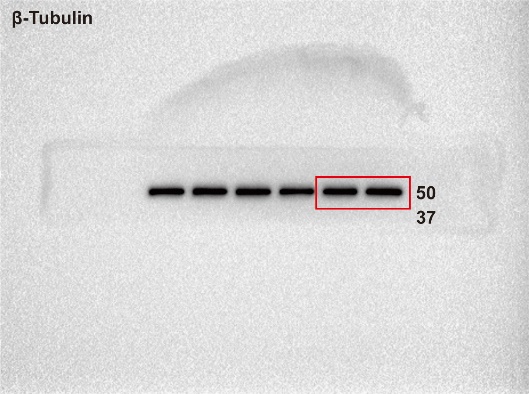
**

**Figure S1J**


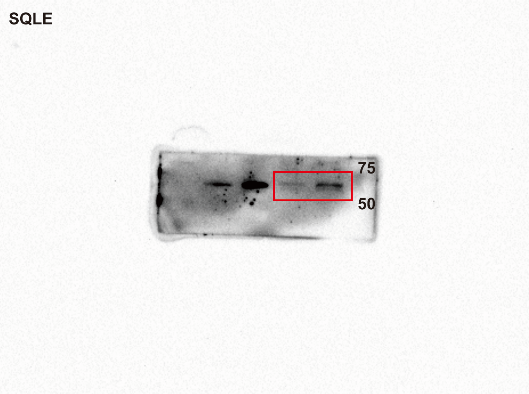
**
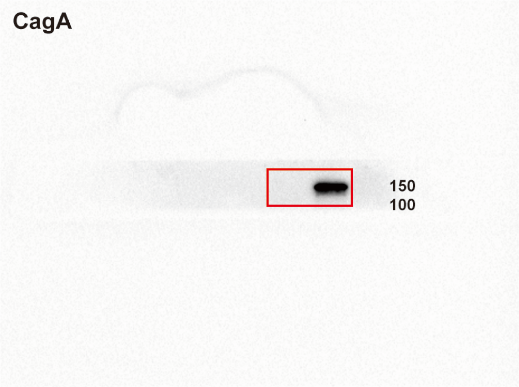
**

**
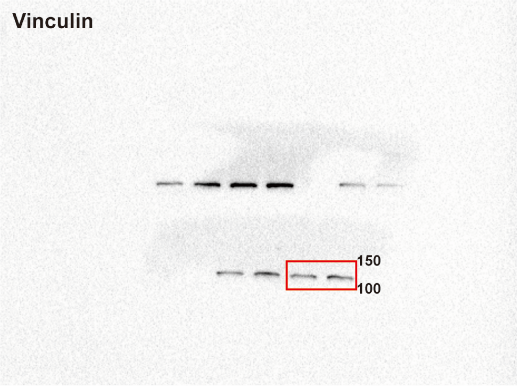
**

**Figure S1L**

**
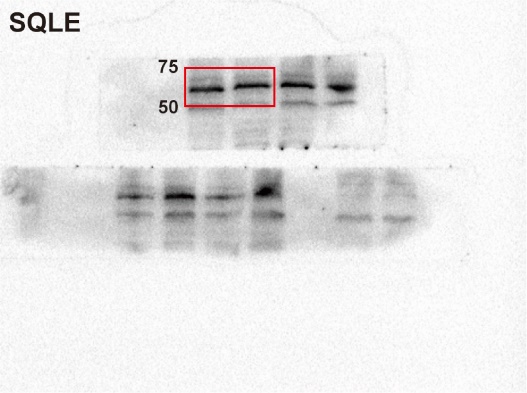

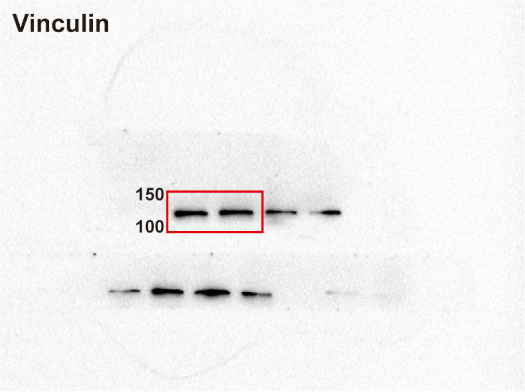
**

**Figure S1N**

**
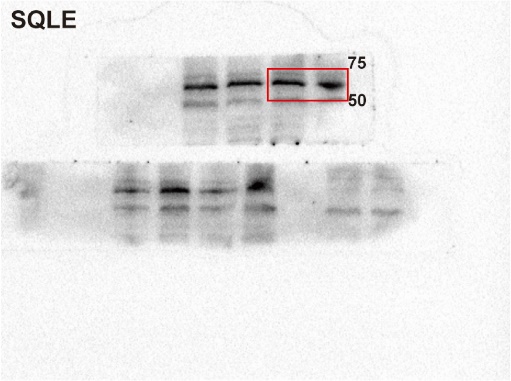
**

**Figure S1P**

**Figure S3B**

**Figure S4B**

**Figure S4C**

**Figure S5A**

**Figure S5B**

**Figure S5E**

**Figure S5F**

**Figure S5G**

**Figure S5H**

**Figure S5J**

**Figure S5K**

**Figure S5L**

**Figure S5M**

**Figure S5N**

**Figure S5O**

**Figure S5P**

**Figure S5Q**
